# Phospholipid Vesicles for Dermal/Transdermal and Nasal Administration of Active Molecules: The Effect of Surfactants and Alcohols on the Fluidity of Their Lipid Bilayers and Penetration Enhancement Properties

**DOI:** 10.3390/molecules25132959

**Published:** 2020-06-27

**Authors:** Hiba Natsheh, Elka Touitou

**Affiliations:** The Institute for Drug Research, School of Pharmacy, Faculty of Medicine, The Hebrew University of Jerusalem, Ein Karem, P.O. Box 12065, Jerusalem 9112102, Israel; hiba.natsheh@mail.huji.ac.il

**Keywords:** phospholipid nanovesicle, transfersomes, ethosomes, glycerosomes, transethosomes, dermal/transdermal, nasal, skin, penetration enhancement, ethanol, edge activator

## Abstract

This is a comprehensive review on the use of phospholipid nanovesicles for dermal/transdermal and nasal drug administration. Phospholipid-based vesicular carriers have been widely investigated for enhanced drug delivery via dermal/transdermal routes. Classic phospholipid vesicles, liposomes, do not penetrate the deep layers of the skin, but remain confined to the upper stratum corneum. The literature describes several approaches with the aim of altering the properties of these vesicles to improve their penetration properties. Transfersomes and ethosomes are the most investigated penetration-enhancing phospholipid nanovesicles, obtained by the incorporation of surfactant edge activators and high concentrations of ethanol, respectively. These two types of vesicles differ in terms of their structure, characteristics, mechanism of action and mode of application on the skin. Edge activators contribute to the deformability and elasticity of transfersomes, enabling them to penetrate through pores much smaller than their own size. The ethanol high concentration in ethosomes generates a soft vesicle by fluidizing the phospholipid bilayers, allowing the vesicle to penetrate deeper into the skin. Glycerosomes and transethosomes, phospholipid vesicles containing glycerol or a mixture of ethanol and edge activators, respectively, are also covered. This review discusses the effects of edge activators, ethanol and glycerol on the phospholipid vesicle, emphasizing the differences between a soft and an elastic nanovesicle, and presents their different preparation methods. To date, these differences have not been comparatively discussed. The review presents a large number of active molecules incorporated in these carriers and investigated in vitro, in vivo or in clinical human tests.

## 1. Introduction

The structure of the skin and nasal membrane regulates the need for using enhanced delivery carriers to overcome these permeability barriers.

The skin is the largest organ of the body and protects it from exogenous materials. The outermost layer of the skin, the stratum corneum (SC), consists of nonviable, anucleate, keratinized cells and forms an effective barrier to retain water within the body and keeps exogenous compounds out. Most therapeutic molecules lack the ability to efficiently cross the skin barrier. Lipid molecules in the stratum corneum, mainly ceramides, cholesterol and fatty acids, play a major role in determining the barrier function of this layer. These lipids are packaged into small organelles to form lamellar granules which fuse in an edge-to-edge manner to form flattened lamellar disks organized in paired bilayers [1]. The main penetration pathway for molecules is the intracellular route through these lipid bilayers of the SC [2]. Viable epidermis and dermis are layered tissues containing a high-water concentration, representing a hydrophilic region for molecules to partition in their way from the SC to the blood vessels.

To overcome the skin barrier layers and allow active molecules penetration, the use of chemical penetration enhancers, molecules that interact with skin constituents to promote drug flux, has presented a long-standing approach. Fatty acids, urea, surfactants, pyrrolidones, dimethyl sulfoxide (DMSO), n-decyl methyl sulfoxide and short chain alcohols are some examples of chemical penetration enhancers used for dermal/transdermal administration of active molecules [3].

Liposomes, the classic phospholipid vesicles, have been shown to carry drugs into the upper layers of the skin. However, the ability of liposomes to facilitate drug penetration into the deep skin layers is limited [4,5]. Over the last three decades, an increasing attention has been given to phospholipid vesicles altered by surfactants and by ethanol to be used for delivery of active agents for dermal/transdermal and nasal administration [4,6,7,8,9,10,11]. These types of vesicular carriers were not investigated for oral or parenteral administration. This could be due to the large fluid volume in these tracts which may interfere with the vesicle initial structure. These vesicles may also be degraded in the gastrointestinal tract and their composition may be toxic when injected in the blood.

Transfersomes [6] and ethosomes [4,7] are the most investigated enhancing penetration phospholipid vesicles. These two innovative systems facilitated abundant research and scientific publications. Small and large active molecules with various lipophilicities were incorporated in these carriers. The systems were investigated for treatment of a wide variety of skin diseases such as microbial and viral infections, inflammation, psoriasis, atopic dermatitis, skin cancer and skin pigmentation disorders. Furthermore, systems containing molecules for transdermal delivery to the systemic circulation were investigated for hormone replacement therapy, hypertension, Parkinson’s disease, diabetes mellitus, hot flushes, hypertension, psychosis and depression. 

Another important drug administration method is the nasal route. It has been traditionally used to treat localized aliments such as allergic rhinitis and symptoms of the common cold. For more than three decades, this route has been explored for drug systemic action, and as an alternative to the oral or parenteral ways. Remarkable advantages including rapid onset, avoidance of first pass metabolism by the liver and high patient compliance were achieved following nasal drug administration [12]. More recently, the possibility of nasal drug delivery to the brain and the central nervous system (CNS) has become an attractive field of research [2,13]. 

The human nasal cavity is about 12 cm long, has a 15 mL volume capacity and a relatively large total surface area of more than 150 cm^2^. The cavity is divided longitudinally into two non-connected parts by the midline cartilaginous bony nasal septum [14]. Each part consists of three main regions: the nasal vestibule, the respiratory region and the olfactory region. While the nasal vestibule is considered a continuation to the skin and has no role in the delivery mechanism, the other two regions control the delivery of nasally administrated drugs [15]. The respiratory region, the largest part of the nasal cavity, is covered with microvilli, which increases the surface area. Furthermore, this region is highly innervated with capillary blood vessels and fibers of the trigeminal nerve. The rich capillary innervation together with the large surface area make this region the major site for systemic absorption [16,17]. The olfactory region is responsible for the olfaction sense and is located at the roof of the nasal cavity where the olfactory nerve ends. Such position makes this nerve unique in that it is the only part of the central nervous system exposed to the peripheral environment. The mucosa in this region consists of three types of cells: supporting epithelial cells, basal cells and olfactory neurons originating at the olfactory bulb in brain and terminating at the olfactory epithelium. The neuronal supply in this region contains also nerve fibers of the maxillary branch of the trigeminal nerve [18]. Following nasal administration, drugs come into contact with the nasal epithelium where a number of concomitant procedures could occur. Part of the administrated dose will be propelled towards the esophagus by mucociliary clearance or eliminated by the enzymatic degradation process [15]. Drugs that have escaped the local elimination can reach the systemic circulation mainly through the respiratory mucosa. An additional possibility is the drug transport to the brain and the central nervous system (CNS) via the olfactory region and the trigeminal nerve pathways. 

Drugs can cross the nasal epithelium by the following routes: (1) paracellular through the tight junctions, (2) passive and active transcellular pathways including carrier mediated transport and (3) the transcytotic route [13,18]. Nasal administration of small lipophilic molecules generally has a good bioavailability. However, barriers in the nasal mucosa contribute to insufficient delivery of hydrophilic drugs of low molecular weight and large molecules such as peptides and proteins. This is explained by the existence of the tight junctions between the intricately connected epithelial cells of the paracellular pathway [16].

Efficacy and applications of transfersomes and ethosomes in the field of dermal and transdermal drug administration have been extensively reviewed [19,20,21,22,23,24,25,26,27,28,29,30].

More recently, carriers containing altered phospholipid vesicles have been investigated for nasal drug administration. The first publications in this field were by Touitou et al. [8,31]. Hussain et al. [32] reviewed elastic liposomes as novel carriers for drug administration via dermal/transdermal and nasal routes. This field has been growing over the past few years in line with the increased attention given to drug delivery via the nasal route.

This specific literature does not often distinguish between the characteristics of the various types of altered phospholipid vesicles. As per our knowledge, the effect of ethanol and surfactant on the structure of each type of vesicle, its mechanism of action and application mode on the skin were not extensively reviewed. 

In this analysis, we emphasize the differences between a soft and an elastic phospholipid vesicle for dermal/transdermal and nasal drug administration. The review focuses on the main characteristics, the specific preparation methods and mechanism of action for each type of vesicle. Numerous active molecules investigated using these carriers are also reviewed. In addition, local safety and tolerability of dermal and nasal administration of these drug delivery systems are covered. We believe this review will help researchers and formulators designing a delivery system to choose the appropriate carrier for a specific molecule based on the type of the nanovesicle they need to use, the product administration mode and manufacture facilities.

## 2. Phospholipid Nanovesicles for Dermal and Transdermal Delivery of Active Molecules 

Various approaches have been proposed to overcome the SC barrier and enhance the delivery of molecules to the deep skin layers and through the skin [2]. Liposomes are one of the first vesicular carriers investigated in this field. These are phospholipid vesicles with a spherical shape consisting of one or more bilayers. Liposomes were considered for the delivery of drugs with various properties. However, the ability of conventional liposomes to enhance drug penetration into the deeper skin regions and to the systemic circulation is minimal. They generally lead to compound accumulation in the SC without being able to facilitate its delivery to the hydrophilic deeper layers [4,5]. In the last three decades, phospholipid vesicles with altered structures have been investigated as carriers for enhanced drug permeation into, or across the skin. Surfactants edge activators and alcohol have been used to generate the deformable and the soft nanovesicles, respectively. 

### 2.1. Types of Phospholipid Nanovesicles for Dermal and Transdermal Drug Administration: The Effect of Alcohol, Edge Activators and Glycerol on Their Properties 

#### 2.1.1. Phospholipid Nanovesicles Containing High Concentrations of Ethanol for Dermal and Transdermal Drug Administration

Ethosomes, composed of phospholipid, ethanol (20–50% *w/w*) with, or without glycols, water and the active molecule, are in general multilamellar nanovesicles containing bilayers from wall to wall of the vesicle. As first introduced by Touitou [7], the ethosomal phospholipid vesicles can contain short chain alcohols like ethanol or isopropyl alcohol with an optional addition of glycols such as propylene glycol and Transcutol^®^. The alcohol high concentration in ethosomes fluidizes the phospholipid bilayers and organizes them in a spherical lamellar-closed shape, as shown by phosphorous (31P) NMR data. This high degree of fluidity for lipid bilayers in this vesicle compared to liposome was confirmed by the results of the fluorescent anisotropy measurements of 9-antrivinyl labeled analogue of PC (AVPC), [4]. The softness of ethosomes was further confirmed by the differential scanning calorimetry (DSC) data of drug-containing and without-drug ethosomes; in these systems, a reduction of up to 30 °C in the main phase transition temperature (Tm) of the phospholipid when compared with liposomes, not containing ethanol but containing the same concentration of phospholipids and the drug, was observed (Table 1).

**Figure 1:**
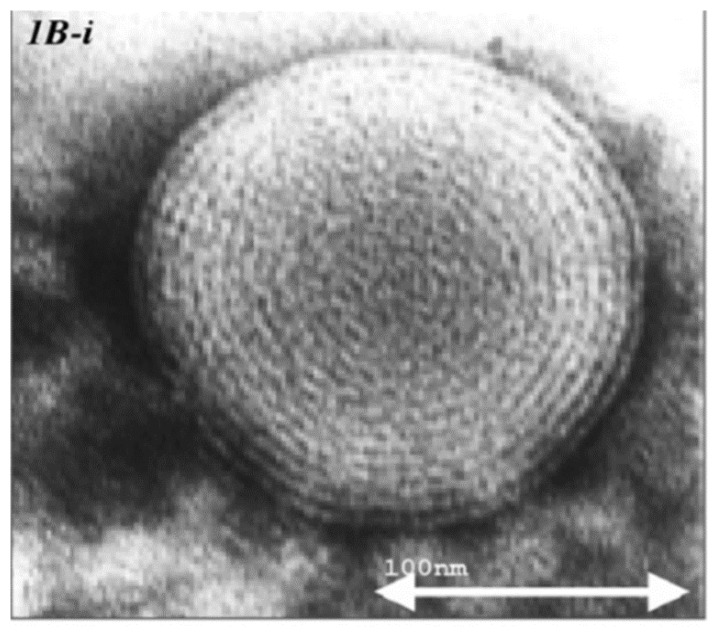
Transmission electron (TE) micrographs of multilamellar to the core minoxidil ethosomes. Reproduced from Reference [24] with permission.

Electron microscopic (EM) micrographs of ethosomes, indicated the presence spherical closed vesicles with lamellae spaced evenly to the core (Figure 1) [4]. While some active molecules, such as minoxidil and testosterone, have not affected the multilamellar structure of the nanovesicles [4,42], the incorporation of erythromycin and buspirone HCl produced uni-lamellar ethosomes [34,43]. Two or three lamellae were observed in trihexyphenidyl (THP) ethosomes [35]. Ethosomes were found to be nanosized vesicles with a narrow and homogenous size distribution. Empty vesicles composed of 2% phospholipid and 30% ethanol showed a mean size distribution of 150 ± 4 nm. By increasing the ethanol concentration from 20 to 45%, the vesicles size decreased to 103 nm. Notably, the corresponding liposomes, prepared by the conventional film forming method, had a larger average size of 677 ± 31 nm [4].

Ethosomal gel systems were also tailored to increase the viscosity and improve the plastic flow of the systems. In these systems, ethosomes were incorporated with mixing in carbomer gels using various types of this polymer at a concentration of 0.25–1% *w/w* and neutralizing bases [34,36,43,44,45]. 

Ethosomal systems were found to be much more efficient in delivering active molecules into and across the skin in terms of quantity and depth when compared to classic liposomes or to hydroalcoholic solutions, containing the same ethanol concentration as the vesicular system. In a 24 h in vitro experiment, the minoxidil amount permeated across excised nude mice skin from non-occluded ethosomal system was 637.0 ± 92.0 µg/cm^2^. This amount is 10, 45 and 35 times higher compared to 2% phospholipid in ethanol, 30% ethanolic solution or absolute ethanol systems, respectively. Ethosomes also enhanced the skin localization of minoxidil two-, seven- and five-fold compared to the above-mentioned control systems, respectively. In another in vitro experiment using Franz diffusion cells and confocal laser scanning (CLS) microscopic examination, calcein incorporated in an ethosomal system penetrated nude mouse skin to a depth of 160 µm compared to only 80 and 60 µm from a hydroethanolic solution and liposomes, respectively [46].

#### 2.1.2. Phospholipid Nanovesicles Containing Surfactants for Dermal and Transdermal Drug Administration

Transfersomes are the first-generation modified liposomes with elastic and flexible properties. They were introduced by Cevc and Blume [6]. These stress responsive nanovesicles were designed to facilitate the penetration through pores much smaller than their size [25]. The special properties of transfersome, distinguishing them from liposomes, are attained by incorporating edge activators, mainly surfactants, into the vesicular membranes in proper ratios [47]. Sodium cholate [48,49], bile salts [50], oleic acid, Span 80 [37], Tween 20 [51], dipotassium glycyrrhizinate [38] and Tween 80 [37,52] are examples of surfactants used for the preparation of transfersomes. In his transfersomal systems, Cevc used surfactants in transfersomal systems at a concentration of 0.02–10% [48,49,53]. These surface-active agents impart deformability and flexibility to the phospholipid vesicle. However, at higher concentrations, these additives may generate mixed micelles instead of vesicles [37].

Liposomes modified with edge activators or vegetable oils have also been investigated by Trotta et al. [38]. Deformable liposomes for improved dermal delivery of methotrexate were prepared. Epikuron 200 (phospholipid containing 95% phosphatidylcholine, PC), or hydrogenated lecithin (PL100H)-based liposomes were modified by adding dipotassium glycyrrhizinate (KG), a natural molecule with emulsifying properties. These modified liposomes showed a high elasticity and deformability that allowed them to pass through barriers with pores smaller than their own diameters by a factor of about three. The size of liposomes containing KG and methotrexate before and after passing through 100 nm pores was reported to be similar. For example, nanovesicles of 352 ± 28 nm mean diameter before filtration exhibited a value of 345 ± 20 nm after filtration. The effect of deformable liposomes on the skin permeation profile of methotrexate was evaluated in vitro on pig ear skin. The cumulative drug amounts permeated through the skin after 24 h from deformable liposomes containing PL100H and PC were 16.8 ± 4.0 and 23.55 ± 4.3 µg, respectively. Classic liposome and aqueous solution had a lower permeation profile and an equal drug amount of 5.7 µg in 24 h.

**Figure 2:**
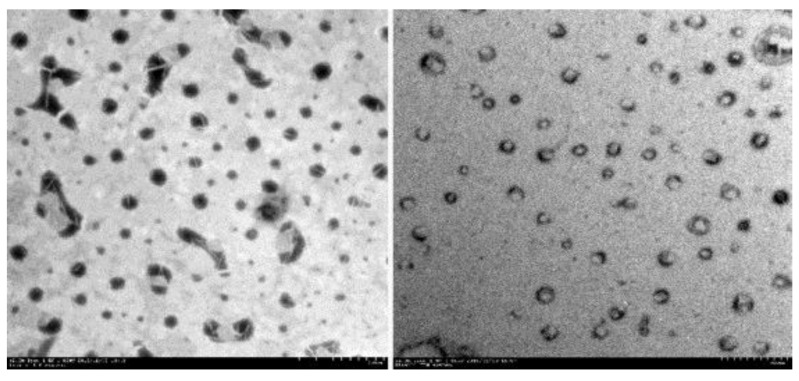
TE micrograph of transfersomes showing hollow nanovesicles. Reproduced from Reference [54] with permission.

Zheng et al. [54] studied the morphology of soybean lecithin (SPC) transfersomes containing sodium deoxycholate as edge activator and itraconazole as an active compound model. Nanovesicles with high stability, composed of 6 mmol/L SPC, 6 mmol/L sodium deoxycholate and 1.23 mmol/L itraconazole, were prepared according to a thin layer evaporation method. The mean size of the obtained nanovesicles was found to be around 100 nm and appeared as tiny hollow vesicles with surrounding darkness, as shown by transmission electron (TE) microscopy (Figure 2).

Notably, early work on transfersomes did not pay attention to the effect of edge activators on the fluidity of the vesicles’ bilayers. In later work, it was shown that the addition of Tween 80, Span 80 and oleic acid to dipalmitoylglycerophosphatidylcholine (DPPC) vesicles reduced the Tm values by 0.84, 7.3 and 2.1 °C, respectively [37]. Furthermore, vesicles containing PL100H and 1% *w/w* KG showed a 0.9 °C reduction in the Tm value relative to liposome (Table 1). This DSC data showing low differences in the Tm values suggest a minor change in the lipid bilayer fluidity of the phospholipid vesicles by the addition of a surfactant. These small changes in the Tm values in transfersomes compared with ethosomes can be due the differences in the concentrations of edge activators and ethanol in transfersomes and ethosomes, respectively. While high ethanol concentrations are used in ethosomes, the percentage of edge activators used in transfersomes is quite small. As mentioned above, surfactants at high concentrations may lead to the destruction of the vesicular structure and may irritate the skin.

Transethosomes are phospholipid carriers derived from ethosomes and transfersomes. They contain high ethanol content (≥20%) together with edge activators. Song et al. [55] reported preparing voriconazole transethosomes by adding edge activators, such as Tween 80, sodium taurocholate and oleic acid at a concentration of 0.53% *w/w,* to ethosomes containing 30% ethanol. Transmission electron microscopic (TEM) examination indicated the presence of irregular-shaped vesicles with a mean size ranging from 146 nm to 192 nm, when compared to the corresponding ethosomes and transfersomes. The irregular shape may be due to the combination of ethanol and edge activators that causes a rearrangement in the lipid bilayer of these vesicles. Transethosomes containing sodium taurocholate and Tween 80 exhibited elasticity indexes, defined by the authors as the fraction of recovered suspension after extrusion through a membrane of 50 nm pore size, of 6.04 and 9.68, respectively. Recently, Fe-chlorophyllin transethosomes based on phosphatidylcholine, edge activators and 20% *w/v* ethanol were prepared and characterized. Tween 80, Tween 20, Span-20 or Cremophor-A25 were used as edge activators at the concentrations of 0.1 and 0.3% *w/v*. The mean vesicles size ranged between 455 to 686 nm when these surface-active agents were used at the lower concentration. The mean vesicles size values decreased to 305–385 nm for 0.3% *w/v* edge activators. Furthermore, vesicles containing 0.1% *w/v* Cremophor A25 exhibited a deformability index (26.2 ± 3.8 mL/s) [56]. 

#### 2.1.3. Phospholipid Nanovesicles Containing Glycerol for Dermal and Transdermal Drug Administration

Manca et al. investigated phospholipid nanovesicles containing glycerol (10–30% *v/v*), which they call glycerosomes. These vesicles also contain cholesterol. The authors claim that this trihydric alcohol may increase the fluidity of the vesicles’ bilayer, thus improving the ability of the carrier to enhance molecule penetration of the skin. It is noteworthy that compared with ethosomes, in which the use of phospholipids with relatively high content of phosphatidylcholine was mostly reported, glycerosomes were prepared using DPPC, hydrogenated soy phosphatidyl choline (PL90H) and 1,2-dimyristoyl-sn-glycero-3 phosphatidylcholine (DMPC) [39,40,41]. 

The reduction in Tm due to the presence of glycerol in glycerosomes, (Table 1) is quite small. DPPC liposomes displayed a Tm of 43.51 ± 0.20 °C. The addition of glycerol (from 10 to 30%) to these liposomes caused a decrease in Tm by 0.5–0.9 °C to reach values of 42.99 ± 0.18 °C for 10% glycerol, and 42.20 ± 0.25 °C when the highest amount of glycerol was used (30%) [39]. This trend was observed again, with glycerosomes containing DMPC in which the addition of glycerol at various concentrations caused small shifts of 0.51 °C in the Tm values from ∼24.98 °C in the vesicles lacking glycerol, to lower temperatures up to ∼24.47 for glycerosomes with maximum glycerol content [41]. A similar shift of 0.5 °C was also reported for empty and diclofenac sodium loaded glycerosomes prepared with PL90H [40]. This effect of glycerol on Tm values is considered low when compared to that of ethanol in ethosomes. It is also possible that cholesterol present within glycerosome bilayers, as a membrane stabilizer, increased their rigidity. This assumption is supported by previous findings, indicating that cholesterol addition to DPPC liposomes resulted in the formation of vesicles with higher Tm values [37]. 

**Figure 3:**
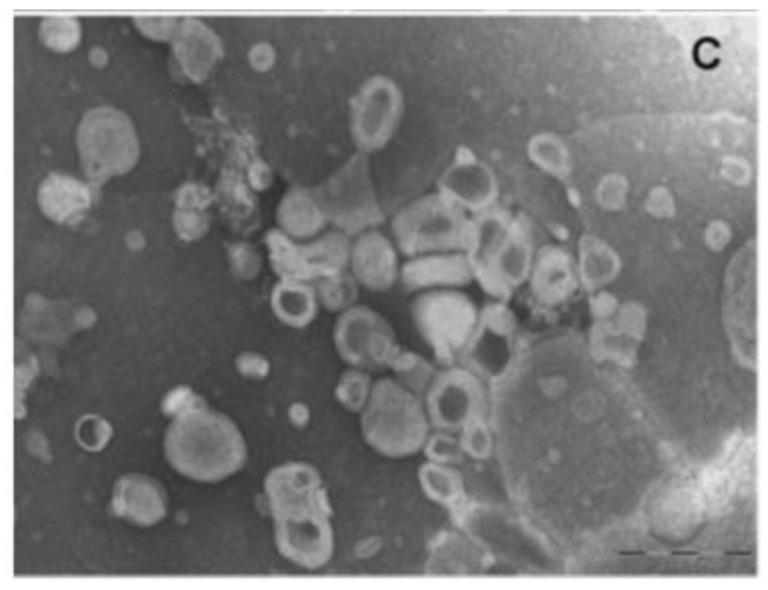
TE micrographs of diclofenac sodium loaded in PL90H-based glycerosomes containing 20% glycerol, scale bar 200 nm. Reproduced from Reference [40] with permission.

Glycerosomes are composed of multilamellar spherical nanovesicles with a mean diameter of 105–130 nm (Figure 3). Regarding the deformability of the vesicular bilayers, as evaluated by extrusion through polycarbonate membrane, glycerosomes containing 10% glycerol were as deformable as the control liposomes. On the other hand, the vesicle elasticity improved as glycerol content increased; empty and drug-loaded glycerosomes with 30% glycerol were two-fold more deformable than the control. This small decrease is similar to the effect of surfactants on the Tm values of phospholipid. 

Diclofenac glycerosomes prepared with DPPC or PL90H displayed a similar penetration behavior when applied in vitro to ear pig skin. These systems containing 20% and 30% glycerol were more efficient compared to systems containing 10% glycerol or to conventional liposomes. Diclofenac mainly accumulated in the SC and in the epidermis [39,40].

### 2.2. Preparation of the Various Types of Phospholipid Vesicles for Dermal and Transdermal Drug Administration

Ethosomes are prepared by a mixing method, in which phospholipids and drugs are dissolved in ethanol at room temperature followed by the addition of propylene glycol, if present. Water is then added slowly in a fine stream, through constant mixing with an overhead stirrer. Another method reported by Touitou [7] is the “hot” method of dispersing the phospholipid in water warmed in a 40 °C bath. In a well-sealed separate vessel, ethanol and optionally propylene glycol are mixed and heated to 40 °C. The organic phase is then added by mixing to the aqueous one. The drug can be dissolved in water or ethanol during the preparation process, depending on its hydrophilic/hydrophobic properties. These preparation methods are environmentally friendly processes and do not involve solvent evaporation [4]. We recommend these methods to prepare ethosomes. Other researchers are using a method which they call “the ethanol injection method” [57]. The described method is a mixing method and is not the procedure described by Batzri and Korn [58] in which the maximum ethanol concentration is 7.5%. A much higher ethanol concentration is needed for the preparation of ethosomes. The thin layer evaporation method [59,60] is also not recommended for the preparation of these systems. Our view is that these procedures which include homogenization and dialysis are not appropriate. In general, by the thin layer method, the phospholipid is solvated by a mixture of ethanol and water/buffer which could affect the entrapment of ethanol between the bilayer. By dialysis, the initial ethanol concertation could be altered (unpublished data). This review does not cover studies on phospholipid vesicles prepared by these methods or those containing low ethanol concentrations [61]. 

The main method of preparation for transfersomes is by using the thin layer evaporation procedure. Lipid soluble components (phospholipids, edge activators and the drug) are dissolved in suitable organic solvents (e.g., methanol/chloroform), then dried under a nitrogen stream and vacuum. This thin film could also be evaporated with a diffusion pump for 12 h. The obtained thin layer is dispersed in an aqueous medium by shaking. This process yields a heterogenous vesicles suspension which is homogenized by ultrasonication [48,51]. Another reported method for transfersomes preparation is by dispersing the lipids in a small amount of ethanol (~10% *v/v* final concentration). The aqueous phase containing the active ingredient is then added. The resulting suspension is frozen and thawed (2–3 times) then sterilized by filtration through a series of microporous filters with decreasing pore size (800–50 nm) to narrow the final size of the vesicles [49,50,62]. 

Some studies reported preparing transethosomes by dissolving the lipid content and the drug in ethanol, followed by adding the aqueous phase [4]. The edge activator is added to the composition at different stages. Song e al., [55] added the surfactants to the final ethosomal system, while Rady et al. [56] dissolved them with the lipid content in ethanol. Other investigations used the thin layer evaporation method to obtain these vesicular carriers [63,64]. 

Glycerosomes were also prepared by thin film hydration method. As reported by Manca et al., [39,41] the lipid content (phospholipid and cholesterol) is dissolved in chloroform. Upon solvent evaporation, the thin layer is hydrated in two steps with glycerol/ phosphate-buffered saline (PBS) and diclofenac sodium salt by mechanical shaking. The final dispersion is then homogenized by ultrasonication. In other studies, the drug and the lipids are dissolved in the organic solvent to form a drug-containing thin layer [40,65]. 

### 2.3. Mechanism of Action of Phospholipid Nanovesicles for Enhanced Dermal and Transdermal Delivery of Active Molecules

**Figure 4:**
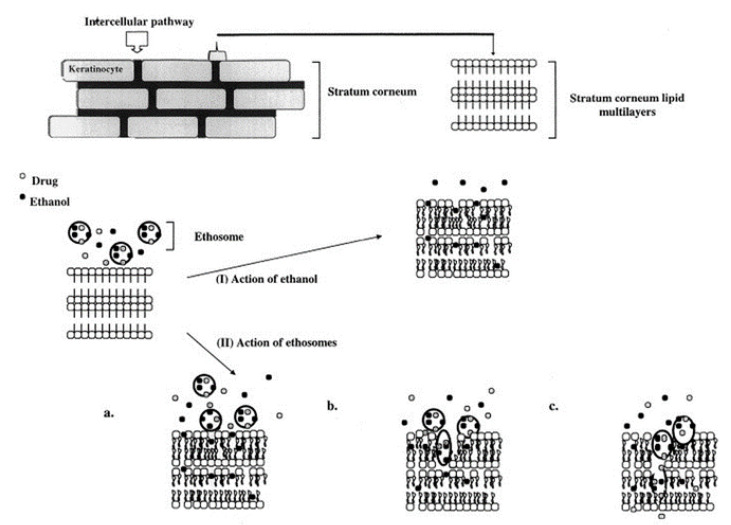
Proposed model for the mechanism of skin delivery from ethosomal systems containing phospholipids, ethanol and the drug. Reproduced from Reference [4] with permission.

In ethosomal systems, ethanol at high concentrations (up to 50%), plays two major roles, fluidization of the phospholipid vesicle bilayers and disruption of the lipids organization in the SC, allowing the vesicles to penetrate deeper into the skin. Enhanced skin penetration and permeation are obtained in comparison with the use of rigid liposomes. Touitou et al. proposed a model to explain the mechanism of action of these soft nanovesicles (Figure 4). According to this model, ethosome application to the skin is followed by a number of concomitant processes involving the stratum corneum pathway. First, the organization of the lipids in the SC is disrupted by ethanol leading to their fluidization. The soft ethosomes are then able to penetrate through the disrupted SC bilayers and reach deeper into the skin where they fuse and release their drug content [4]. The authors suggest that ethosomes enhance molecule penetration through the skin mainly via the intracellular pathway. This hypothesis was supported by the enhanced in vitro delivery into fibroblasts of the amphipathic probe 4-(4-diethylamino) styryl-*N*-methylpyridinium iodide (D-289), the lipophilic probe rhodamine red (RR) and fluorescent phosphatidylcholine (PC*), as examined by confocal laser scanning microscopy (CLSM) and fluorescence-activated cell sorting (FACS). The measured RR fluorescence intensities by CLSM were 150, 40 and 20 arbitrary unit (A.U.) for the probe delivered in ethosomes, hydroethanolic solution and liposomes, respectively. The data generated from FACS examination indicated that only ethosomes yielded a visible penetration of the incorporated probes. It was shown that the intracellular delivery patterns differed between the three probes. D-289 and RR diffused and distributed throughout the cell, including the nucleus, with RR highly concentrated in the outer membrane. The labeled phospholipid, PC*, was observed only in the cytoplasm of the cells [46]. 

The above mechanism theory is sustained by the work of Mbah et al. [66] which investigated the mechanism of action of ethosomal gel containing griseofulvin by measuring the Tm of treated rat skin by DSC. The obtained thermogram displayed a reversible endothermic peak at 69.9 °C which continued to an enlarged peak at 94.0 °C. This could be attributed to the melting and fluidization of the fatty acids and keratin proteins in the SC corneocytes. This disordering of the skin layers, including fluidization of the intercellular spaces, possibly caused by the ethosomes, seemed to loosen the lipid bilayer, allowing easier diffusion into the deep skin layers. 

The effect of occlusion on the permeation profiles of fluorescein isothiocyanate (FITC)–bacitracin ethosomes applied to human cadaver skin in Franz diffusion cells was studied by Godin and Touitou [33]. Transport kinetics of the labeled active ingredient showed flux values of 0.34 ± 0.03 and 0.29 ± 0.02 mg/h cm^2^, for the occluded and nonoccluded diffusion cells, respectively. These flux values indicate that occlusion had almost no effect on skin permeation of FITC–bacitracin from ethosomes.

In the first publication on transfersomes, Cevc and Blume proposed and investigated the mechanism of action of these carriers. They hypothesized that the osmotic gradient between the skin surface and its deeper layers, which is created by the difference in total water concentration between the two regions, can enforce the vesicles flow into and through this barrier. The results indicated that these driving forces are sufficiently strong to push more than 0.5 mg of transfersomes per hour and cm^2^ through the SC. However, this effect did not enhance vesicles penetration into the deeper skin layers. The authors reported that the efficiency of transfersomes as penetration enhancing carriers through the skin was highly affected by occlusion. Their non-occlusive application, where water evaporation dehydrates the vesicles, enabling penetration of great quantities of applied lipids into the deeper skin layers. Less than 50% of the total dose applied non-occluded remained confined to the skin surface as compared, with more than 87 % in the case of occlusive application. The authors claim that the use of transfersomes under non-occlusive conditions is mandatory to permit water evaporation from the formulation and maintain this hydration gradient [6].

**Figure 5:**
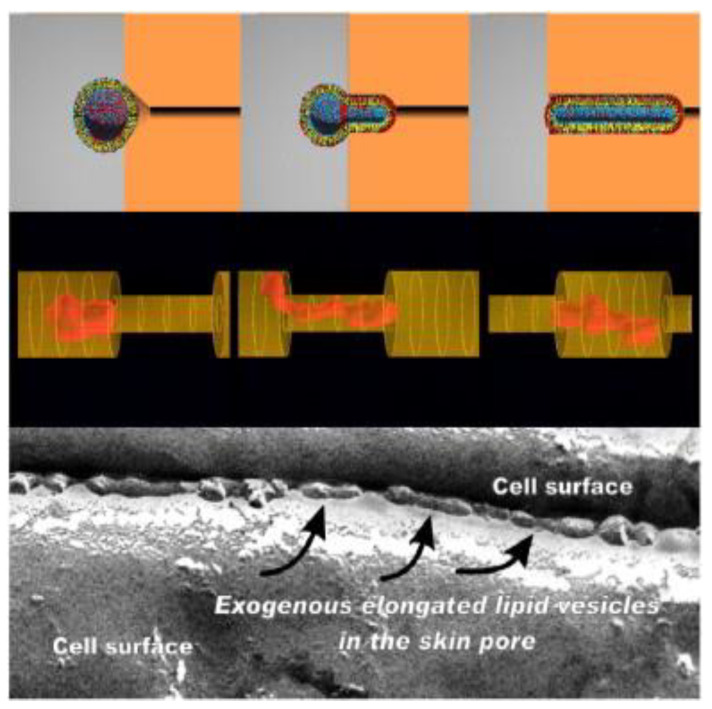
Proposed mechanism of skin penetration by transfersomes. Top: computer simulated distribution of more (red, e.g., a surfactant) or less (blue, e.g., a phospholipid) water soluble molecules with hydrophobic (yellow) chains arranged in a mixed amphipathic spherical bilayer as a function of predefined vesicle shape. Middle: a simulation of a highly deformable, infinitely permeable, non-destructible vesicle forced by a horizontal gradient into a pore with 0.5 smaller diameter. Bottom: an electromicrograph of elongated, deformable vesicles in an inter-corneocyte water-filled channel within the human stratum corneum after an application of a lipid preparation on its open surface. Reproduced from Reference [68] with permission.

To understand the different routes of penetration into the SC by means of transfersomes, vesicles prepared with rhodamine labeled phospholipids were applied nonoccluded to mice skin in vivo and ex vivo. Treated skin CSL micrographs, showing a bright cobweb, showed that transfersome-mediated transport is confined to the intercellular pathway. Two quantitatively different hydrophilic pathways were suggested: a strongly tortuous intercluster route with low penetration resistance between groups of corneocytes and an intercorneocyte pathway that goes between the individual corneocytes within the cell clusters [48]. Cevc and colleagues proposed that transfersome flexibility and elasticity achieved by edge activators enable them to adjust their shape and penetrate the dense SC layer, which contains very small “pores” relative to the vesicle diameter [32,67,68]. The simulated model in Figure 5 (upper and middle panels) shows the vesicles deformability during the passage through the skin. The nonoccluded-applied deformable vesicles penetrate the stratum corneum and are visible in the stratum cornuem–viable epidermis junction. The electromicrograph (Figure 5 lower panel) indicates the presence of these vesicles in the channel-like structures between the corneocytes [68]. Less deformable vesicles such as conventional liposomes lack the ability to squeeze between the skin layers and therefore stay confined to the skin surface resulting in much lower penetration enhancement [47].

Considering the above, each type of altered phospholipid vesicle enhances the skin penetration by a different mechanism. The high ethanol concentration in ethosomes generates a soft vesicle by fluidizing the phospholipid bilayers, allowing the vesicle to penetrate deeper into the skin. Edge activators contribute to the deformability and elasticity of transfersomes, enabling them to penetrate through pores much smaller than their own size. Table 1 shows minimal differences in Tm values for transfersomes, not exceeding 7.4 °C, compared to 26.4 °C due to a much higher effect of ethanol in ethosomes. It can be assumed that the distinguishing properties between the two types of phospholipid vesicles are their softness, or high deformability and elasticity, respectively. Another important difference between the two nanocarriers is the mode of application on the skin. Ethosomes can enhance skin permeation following either occluded or non-occluded application, whereas a non-occluded application of transfersomes is mandatory to achieve sufficient drug penetration into the SC.

The mechanism of action of transethosomes is not yet well elucidated. Song et al. suggested the presence of a synergic permeation enhancing effect of ethanol and edge activators [55]. 

Glycerosomes, vesicular carriers altered by a trihydric alcohol, appear to have a different mechanism of action from ethosomes. As above mentioned, glycerol slightly lowered the Tm of phospholipid bilayers. Interestingly, the deformability index of these vesicles was increased by at least 10%. A possible mechanism of penetration into the skin involves the deformability and elasticity of these vesicles, similar to transfersomes.

Based on the skin penetration results, glycerol concentration in glycerosomes proportionally affected their ability to increase diclofenac localization in the upper skin strata [39,40,41]. In this work, the effect of glycerosomes was not compared with that of a glycerol solution. Moreover, it was suggested that glycerol presence in the extracellular SC lipids may increase the mobility of ceramide headgroups and promote the fluidity in the hydrophobic regions of the bilayers [69]. 

In a recent study, it was suggested that glycerosomes enhance the permeation of fisetin by fluidizing the lipid bilayers in the skin. DSC examination of rat skin treated with glycerosomes loaded with the drug in vitro indicated a disappearance of a Tm peak at 45.12 °C that was detected in normal untreated skin. The authors explained these structural changes in the skin were caused by lipid disruption and reorientation and increased fluidity of SC lipids. It was hypothesized that glycerol present in glycerosomes induced hydration of the skin and thereby enhanced the fluidity of the lipid bilayer by interacting with the polar head groups of the lipids for easy diffusion of the formulation [65].

### 2.4. Active Molecules Incorporated in Phospholipid-Altered Vesicles Investigated for Dermal and Transdermal Administration

#### 2.4.1. Delivery Properties of Ethosomes Incorporating Various Active Molecules for Dermal and Transdermal Administration

These phospholipid nanovesicular carriers have been investigated for enhanced dermal and transdermal delivery of various active molecules for the treatment of a wide variety of diseases and disorders including herpes infections, acne, erectile dysfunction, skin cancer, hypogonadism and rheumatoid arthritis (Table 2) [4,7,70,71,72].

Ethosomal drug systems were evaluated for their delivery efficacy in numerous in vitro, in vivo in animal models and in clinical studies (Table 2).

The first clinical evaluation of ethosomal-based systems was a double-blind, crossover two-armed, randomized study conducted on 40 subjects with recurrent herpes labialis. The study aimed to test the efficacy 5% acyclovir in ethosomes for the treatment of recurrent herpes labialis compared to the commercial cream (Zovirax^®^) containing an equal amount of the drug and to empty ethosomes. In the parallel arm of the study, the lesions crusting time in the group treated with ethosomal acyclovir was 1.6 vs. 4.3 and 4.8 days for the commercial product and an empty vehicle, respectively. The data in the crossover arm showed lesions crusting periods of 1.8 and 3.5 days for the ethosomal system and the commercial cream, respectively. The crusts disappeared after 4.2, 5.9 days of treatment with acyclovir ethosomes and the commercial cream, respectively. An improvement in the remaining clinical parameters (such as the proportion of abortive lesions) was reported in 30% of episodes treated with ethosomes in both study arms, in contrast with only 10% of episodes treated with Zovirax [73].

Trihexyphenidyl HCl is an anti-muscarinic molecule, used as adjunctive treatment for Parkinson’s disease. The THP ethosomal system was characterized and tested for skin penetration in comparison with a classic liposome. In vitro drug penetration in nude mouse skin pf THP ethosomes was 87, 51 and 4.5 times higher than that of liposomes, phosphate buffer and a hydroethanolic solution, respectively (*p* < 0.01). The quantity of THP that remained in the skin after the 18 h experiment was found to be significantly higher for ethosomes (586 ± 77 µg/cm^2^) compared to liposomes and hydroethanolic solution, which yielded equal values of 416 ± 27 and 415 ± 2 µg/cm^2^, respectively [35]. 

The transdermal delivery of cannabidiol (CBD) incorporated in ethosomes was investigated in nude mice. Upon application of the ethosomal system to the abdomen of Swiss albino (ICR) mice for 72 h, steady-state levels were achieved after around 24 h and lasted at least until the end of the experiment, at 72 h. Furthermore, transdermal application of ethosomal CBD prevented the inflammation and edema induced by a sub-plantar injection of carrageenan in mice [44].

The ethosomal erythromycin system was investigated as a novel approach to treat deep skin and soft tissue bacterial infections by local drug application. Cytotoxicity tests showed that the erythromycin ethosomal system was non-toxic to cultured 3T3 dermal fibroblasts. Susceptibility studies on three bacterial strains (*B. subtilis* ATCC6633, *S. aureus* ATCC29213 and *S. aureus*) showed significantly larger inhibition zones and reduced erythromycin minimum inhibition concentration (MIC) for the ethosomal system compared to erythromycin in the hydroethanolic solution. In vivo application of the ethosomal system to ICR mice skin, previously inoculated with *S. aureus* ATCC29213, resulted in a complete inhibition of the infection. These findings show that ethosomes are efficient tools to carry the antibiotic into deep skin strata for the eradication of staphylococcal infections. On the other hand, treatment with a hydroethanolic solution of erythromycin was not efficient, as deep dermal and subcutaneous abscesses developed. The histopathological examination found that necrosis destroyed skin structures and dense infiltrates of neutrophils and macrophages [34].

Another molecule that was successfully absorbed from ethosomes is testosterone. It is well known that the physiological decrease in the levels of this hormone in men, leads to undesired effects. Occlusive application of a testosterone ethosomal patch to mice skin in vitro resulted in 30 times greater drug permeability than the commercial patch Testoderm^®^, achieving the values of 848.16 ± 158.38 vs. 27.79 ± 16.23 mg, respectively [4]. Furthermore, Ainbinder and Touitou proposed a testosterone replacement therapy approach using the ethosomal system. The systemic absorption of the drug applied from ethosomes was measured in rats and was compared with a commercial gel (AndroGel^®^) following a single dermal application of a 400 mg formulation containing 1% *w/w* drug concentration. The results of blood samples showed Cmax levels of 1970 ± 251 ng/dL and area under the curve (AUC) values of 9313 ± 385 ng.h/dL for the ethosomal system. The commercial product yielded significantly lower Cmax and AUC values of 601 ± 88 ng/dL and 5678 ± 719 ng.h/dL, respectively. The vitro permeation assessment through human skin was performed to establish testosterone skin permeation behavior. The amount of testosterone permeated through the skin after 24 h from ethosomes was 594.57 ± 39.9 µg, this value was six times higher than the amount permeated from the tested commercial product [42].

Ethosomal system containing ammonium glycyrrhizinate, a natural anti-inflammatory agent for acute and chronic dermatitis, was evaluated in a clinical study. The anti-inflammatory activity of the drug from ethosomes was evaluated using a model of skin erythema in twelve healthy volunteers. The participants were treated with 200 µL formulations containing 0.3% *w/v* ammonium glycyrrhizinate in ethosomes versus hydroethanolic and aqueous solutions. Methyl nicotinate was used to induce erythema five hours before the treatments at six sites on the ventral surface of the volunteers’ forearms. The erythema index (ΔEI), measured using a reflectance visible spectrophotometer, was 29.6% for the ethosomal system versus 62.7 and 60.7% for the comparative ethanolic and aqueous solutions, respectively [70].

The effect of clindamycin and salicylic acid ethosomal gel for the treatment of acne vulgaris was evaluated by Touitou et al. [79] in an 8-week randomized double-blind trial on 40 patients. The results showed a significant reduction in the number of inflammatory, non-inflammatory and total lesions in patients with mild to moderate symptoms. In addition, partial, or complete improvement were reported by 71% of the participants. Moreover, 14 out of the 17 participants with a history of previous topical treatment (1% clindamycin lotion, 5–10% benzoyl peroxide gels, 5% benzoyl peroxide–2% erythromycin gel) reported that the ethosomal gel was tolerated much better and caused less erythema, burning, pruritus, and photosensitivity reactions compared to prior topical medications.

Shumilov and Touitou proposed transdermal delivery of buspirone via ethosomes for the treatment of menopausal syndrome. Pharmacokinetic data in rats following transdermal administration of the system indicated that buspirone was present in plasma for 12 h, reaching a Cmax value of 120.07 ± 86.97 ng/mL after 2 h. A relative bioavailability value of 0.89 was calculated for transdermal drug administration vs. oral. The effect of transdermal buspirone on hot flushes was evaluated in ovariectomized (OVX) rats by monitoring changes in the tail skin temperature (TST). The results showed that an alleviation of 1.6 ± 0.7 °C in the TST of OVX rats started 3 h post-buspirone administration and lasted for a further 3 h. Moreover, the anxiolytic effect of the ethosomal drug system was evaluated in a T-maze anxiety rat model 4 and 12 post-treatment. The avoidance latency at the 12 h timepoint was significantly shorter in the buspirone transdermal group (20 ± 118 s) than in the control group treated orally (576 ± 42 s), indicating an efficient anxiolytic effect of the transdermal system [43].

In a pilot clinical study carried out on 16 men with 17 episodes of erectile dysfunction, an ethosomal prostaglandin E1 system was applied on the glans penis. Single topical application of the drug ethosomal system enhanced penile rigidity and improved peak systolic velocity, with erection duration ranging between 10 to 60 min in 12 patients out of 15. On the other hand, an empty ethosomal vehicle did not yield any erectile response or changes in penile blood flow [78].

An interesting study by Paolino et al. tested the in vitro effect of paclitaxel ethosomes on the human squamous-cell-carcinoma line (DJM-1). The importance of drug encapsulation in the nanovesicles was investigated in comparison with its physical mixing with the carrier. In vitro percutaneous permeation profile through human skin revealed a dermal paclitaxel accumulation after 24 of 103.5 µg/cm^2^ when entrapped in ethosomes versus 20.35 and 4.31 µg/cm^2^ for a physical mixture and hydroethanolic solution, respectively. Furthermore, paclitaxel entrapped in ethosomes exhibited efficient anti-proliferative activity in DJM-1 cells. For example, the administration of entrapped paclitaxel at a drug concentration of 0.5 μM led to a cellular mortality of ∼40% of the cultures after 48 h. This value is ∼2.2-fold higher in comparison to the drug effect applied in the physical mixture at the same drug concentration [84].

Chandra et al. investigated an ethosomal gel of methotrexate and salicylic acid for the treatment of imiquimod-induced psoriasis in mice. The psoriasis area and severity index (PASI) score and histopathological examination were performed to evaluate the anti-psoriatic effect of the ethosomal system. At the end of 24 h of the ex-vivo penetration study on pig ear skin, the ethosomal system exhibited prolonged release and retention of methotrexate, 26.13 ± 1.61% and 43%, respectively, in comparison with 6.97 ± 0.06% and 13%, respectively, for the drug solution. The results of the in vivo experiment indicated that after 7 days of administration of the methotrexate-salicylic acid ethosomal system, a maximum PASI was achieved. Normal skin with very mild keratosis was observed in the group treated with the ethosomal system, while a blank gel treated group exhibited signs of moderate to high hyperkeratosis as well as orthokeratosis [107].

Ethosomes have also been investigated as carriers for dermal delivery of natural bioactive molecules. These molecules and their applications are listed in Table 2, and they have been extensively discussed in a recent review by Natsheh et al. [29]. 

Paeonol is a naturally occurring compound with anti-inflammatory, anti-diabetic and pain-relieving activities. A paeonol-loaded ethosomal system with 84.33 ± 1.34% encapsulation efficiency was prepared by Ma et al. In vitro transdermal permeation through rat skin for paeonol applied in ethosomes was 138.58 ± 9.60, compared to only 83.02 ± 10.30 µg/cm^2^ of the control hydroethanolic solution. Skin deposition of the molecule at the end of the permeation experiment was 135.14 ± 15.2 µg/cm^2^ for ethosomes and 52.60 ± 7.90 µg/cm^2^ for the control solution (*p* < 0.01) [106].

Recently, ethosomes containing the anti-gout drug febuxostat were incorporated in hydroxypropyl methylcellulose (HPMC) gel. In vitro skin permeation through a semipermeable cellophane membrane showed that the cumulative febuxostat permeated amount ranged from 43.33 ± 5.3 to 82.14 ± 5.8% by various ethosomal formulations, compared to 49.42 ± 3.29% from the control gel. As measured in the in vivo pharmacokinetic study on rabbits, Cmax values of 49.27 ± 3.19, 35.14 ± 2.52, and 28.26 μg/mL were achieved by the ethosomal system, control gel and oral suspension, respectively. The calculated AUC_0-∞_ was 1009.495 ± 81.24, 788.430 ± 60.20 and 260.028 ± 75.28 μg.h/mL for the above three systems, respectively. Based on these results, the authors suggested this ethosomal system as a promising method for a better management of gout [109]. 

In another recent work, the protective antioxidant effect of coenzyme Q10 incorporated in ethosomes was evaluated in fibroblasts and 3D-reconstituted human epidermis. The results showed that this pretreatment counteracted the H_2_O_2_-induced oxidative stress and prevented the formation of the oxidative damage biomarker 4-hydroxynonenal protein [110].

The data of the above reviewed publications confirm the ability of ethosomes to enhance drug delivery into the deep skin layers and across the skin to the systemic circulation. A wide range of active hydrophilic and lipophilic small and large molecules were successfully delivered by ethosomes. This was shown by experiments carried out in vitro, in vivo and in human clinical studies.

#### 2.4.2. Delivery Properties of Transfersomes Incorporating Active Molecules Investigated for Dermal and Transdermal Administration

As mentioned above, transfersomes with a flexible and deformable structure were evaluated for the delivery of dermal- and transdermal-administrated active molecules. These carriers have been extensively investigated over the last three decades for the treatment of a wide range of disorders, such as diabetes, inflammation, immunization, alopecia, hypertension, depression and skin cancer. In vitro studies on skin and synthetic membranes and in vivo experiments on animal models of these diseases were conducted to test the efficacy of these systems. In addition, transfersomal systems were tested in some clinical studies (Table 3). 

Systemic drug delivery by means of topical administration of transfersomes started early after their development with insulin as large-molecule active ingredients [49]. A transfersulin and transfersomal system containing a rapidly acting human recombinant insulin was evaluated in mice and in a clinical study on healthy volunteers. In mice, the effect was compared with an equal insulin dose of an ethanolic 10% PC solution, PC-based liposomes, and PC-bile salts mixed micelles. Blood glucose and insulin levels were measured during the following 12 h as indicators of the hypoglycemic effect of the drug from each tested system. The results showed that epicutaneous administration of transfersulin decreased the blood glucose levels by 20–30% within 2–4 h. On the other hand, insulin application from the controls as well as empty transfersome did not show any significant reduction in the peptide serum levels over the investigated time period. In the clinical part of the study, the hypoglycemic effects of transfersulin, insulin applied from the above control systems and subcutaneous injection were measured in healthy volunteers. Insulin preparations were uniformly spread on the inner side of one forearm at a dose of 0.6 U/kg. As reported by the authors, only insulin applied in transfersomes yielded a significant systemic hypoglycemic effect. Notably, the onset of action for transfersulin composition was 90–180 min, which is 45–145 min longer than that of subcutaneous insulin.

Nonsteroidal anti-inflammatory drugs (NSAIDS) were among the first active molecules investigated with transfersomes. A lotion-like transfersomal system loaded with diclofenac sodium and transfenac was prepared and investigated by Cevc and Blume [115]. The authors proposed that the association of diclofenac with the ultradeformable carrier could improve its skin deposition and analgesic effect. Transfenac increased by up to 10 times the drug concentration in tissues under the skin of the 3 tested species (mice, rats and pigs) in comparison with diclofenac administration of the commercial hydrogel. For example, in the experiments on rats, transfenac was administrated on the hind leg, and the intramuscular drug concentration was measured at different depths after 12 h. The data showed a diclofenac concentration ranging between 0.5–20 µg/g when administrated at a dose of 0.25–2 mg/kg. Hydrogel administrated at a much higher dose range (1.25–10 mg/kg) created drug levels of only 60.5 µg/g in the tested tissue. In pigs, a diclofenac dose of 0.3 mg/kg applied topically in the highly deformable vesicles was shown to be three times more efficient in terms of drug intramuscular accumulation than drug administration of the commercial gel applied at a dose eight times higher.

Ketoprofen is another nonsteroidal anti-inflammatory drug (NSAID) applied in transfersome gel (IDEA-033). The efficacy of this system was evaluated in comparison with a ketoprofen-free vehicle (TDT 064) in a 12 week randomized study conducted on knee osteoarthritis (OA) patients (n = 866) at 31 centers in Europe. Patients were randomly grouped and treated twice daily for 12 weeks with topical IDEA-033 containing 100, 50, or 25 mg ketoprofen, or TDT 064. The outcomes of this evaluation showed that in all four groups, at least a 50% decrease in pain expression was scored. According to the Western Ontario and McMaster Universities (WOMAC) Osteoarthritis Index, the higher ketoprofen doses (100 and 50 mg) showed a reduction in pain score versus empty vehicle. However, the WOMAC function subscale score did not show a statistically significant superiority of the IDEA033 groups versus TDT 064, with an average improvement of about 40% across all treatment groups. Data analysis according to the patient global assessment score showed that only the 50 mg drug dose was found to be superior compared to the TDT 064 group (*p* = 0.0283). Although responder rates were high in all groups, including the drug free group (100 mg: 88.6%; 50 mg: 86.8%; 25 mg: 88.6%; and TDT 064: 77.5%), the ketoprofen-containing systems showed significantly higher rates versus the group receiving the empty vehicle. Overall, the data showed only a marginal superiority for 50 and 100 mg drug doses of IDEA-033 compared with TDT 064 in reducing knee OA pain [122].

Triamcinolone acetonide (TAC), dexamethasone and hydrocortisone are glucocorticosteroids that were incorporated in transfersome-deformable lipid vesicles and investigated in vitro, in vivo and clinical studies. Aiming to assess equipotent doses of TAC in transfersome and in conventional formulations, a clinical double-blind placebo-controlled trial was performed on healthy volunteers. The biologically effective doses of TAC in transfersome were tested using the UVB erythema test and the skin blanching assay in order to draw comparisons with topical commercial products containing TAC. In addition, the kinetics of action of the various systems were compared. As measured by erythema suppression, a drug dose of 2.5 µg/cm^2^ incorporated in transfersome was bioequivalent to a 10-fold higher dose when TAC was applied in commercial formulations. The authors claim that TAC application from these vesicles was not only more potent, but also caused less side effects. Ultrasonic measurements revealed a significantly reduced skin atrophy rate; the difference between placebo treatment and TAC transfersome at week 2 was nonsignificant. Contrastingly, using TAC cream, the difference was significant at the same time point. A reduction of 12.1% in skin thickness was measured for TAC transfersome, while the bioequivalent dose in TAC cream achieved a 21.1% reduction after a 6 weeks treatment period, indicating the relative safety of the proposed treatment [117].

Transfersomes loaded with central-acting drugs present an interesting field of research. Gupta et al. [121] tested transfersomes for transdermal delivery of the poorly soluble anti-psychotic drug, sertraline. The ex-vivo permeation through rat skin of sertraline from transfersomal gel was compared with transfersomal suspension, control gel, and drug solution. It was found that vesicular gel resulted in a significantly higher (*p* < 0.05) cumulative amount of drug permeation (2.93 ± 0.11 mg/cm^2^) with a lower lag time than the drug solution and drug gel. In vivo studies using the forced swim model test in mice revealed that the transfersomal gel had better antidepressant activity with 0.323 min immobility compared to 2 min in the group treated with the control gel. 

Transfersomal-based systems were proposed as a promising approach to deliver large molecules for immunization purposes. Non-invasive immunization through the skin by means of these systems can lead to a similar or even a slightly higher rise in the antibody titer than subcutaneous injections. For example, transferosome loaded with gap junction protein and human serum albumin resulted in a higher IgA/IgG ratio in the blood than repeated immunogen injections in mice [50]. More recently, Chopra and Cevc immunized mice epicutaneously with a transfersome loaded with tetanus toxoid and monophosphoryl lipid A adjuvant. The results showed that this vaccination approach protected 100% of the tested mice against lethal parenteral tetanus toxin challenge. The authors found that the immune response was mainly achieved from the circulating IgG1 and IgG2b antibody isotypes, indicating a specific Th2 cellular response. Subcutaneous injections of the toxoid also yielded a similar degree of protection, but by different mechanism in which IgG1, IgG2b and IgG2a antibodies indicated a mixed Th1/Th2 response [62].

As mentioned above, transfersomes were evaluated for the delivery of anti-cancer drugs [38,123,126,127]. In a representative example, cisplatin and imiquimod transfersomal formulations were developed to achieve a synergistic effect for the treatment of cutaneous epithelial malignancy. In vivo skin localization of the fluorescence probe, 6-carboxyfluorescin (6-CF), was achieved following topical administration of drugs loaded in transfersomes. On the contrary, intraperitoneal (i.p.) administration of the two molecules led to probe accumulation in other organs (i.e., liver, kidney and gall bladder). The in vivo evaluation of the anti-cancer activity of topical drug combination administrated from transfersomes showed less than 20% incidence of papilloma. Topical application of the drug combination in a solution was much less effective with more than 60% papilloma incidence [123]. 

Transfersomal-based carriers were also evaluated for the transdermal delivery of the anti-hypertensive drugs: felodipine [125], timolol maleate [131,132] and eprosartan mesylate [133]. Morsi et al. [132] measured plasma levels of timolol following non-occlusive single administration of optimized timolol maleate transfersomes to rats in comparison with oral suspension of the drug. As measured by HPLC, oral timolol administration yielded a Cmax of 29.9 ng/mL after 1.5 h. On the other hand, the drug vesicular gel led to a lower Cmax of 10.53 ng/mL after 24 h but prolonged the drug concentration in plasma for over 72 h. The mean AUC_0 to 24_ and AUC_0 to ∞_ for timolol applied topically from transfersomes were increased 4.45- and 3.39-fold compared to oral administration, respectively. In another in vivo study, the anti-hypertensive effect of the topical transfersomal gel application of the angiotensin II receptor blocker, eprosartan mesylate, combined with a commercially available microneedle roller (Dermaroller^®^) was evaluated. Methyl prednisolone acetate was administrated subcutaneously (20 mg/kg/week) for 3 weeks to induce hypertension in rats. The results indicated prolonged and better management of hypertension after the application of transfersomal gel to hypertensive rats pretreated with the Dermaroller when compared with the oral control formulation. This enhanced effect was further confirmed by the decreased levels of angiotensin II type-1 (AT1) mRNA and the AT1 receptor (AT1R) in the aorta smooth vascular muscles of the treated hypertensive rats, as measured by real-time polymerase chain reaction (RT-PCR) and Western blot analysis, respectively [133].

The treatment of alopecia using transfersomal vesicles loaded with minoxidil and caffeine has been studied by Ramazani et al. [52]. The effect of topical application of the drug-loaded transfersomal system to rats for 30 days on hair growth was evaluated in comparison with a transfersome placebo, an aqueous solution of minoxidil and caffeine and a commercial minoxidil hydroalcoholic solution. Ten days after topical administration of the drug combination in transfersomes, fast hair growth, in terms of length and weight, was observed. The aqueous and hydroalcoholic solutions were also shown to increase hair growth, but at a weaker extent. This reported pattern reoccurred 20 and 30 days after topical administration, with more evident effects of minoxidil–caffeine transfersomes in the first 10 days.

A transfersomal gel containing adapalene and vitamin C as a combination therapy for management of this condition has been recently developed [140]. The results of in vivo skin irritation potential, performed on healthy albino rats, showed that transfersomal gel exhibited no irritation symptoms over 72 h and after exposure to sunlight. This was compared with the marketed gel which showed mild irritation redness and erythema. Such reduction in these side effects of adapalene could be due to its entrapment in the vesicles preventing direct exposure to UV radiation. Another suggested possible reason is vitamin C protection against these rays. Treatment efficacy was evaluated in rats with testosterone-induced acne following 4 weeks of the therapy, producing 50 ± 11 papules. The outcomes of this in vivo evaluation indicated a decrease in the acne lesions by more than 65% with a papule count of 14 ± 8 due to transfersome-containing adapalene and vitamin C. A papules count of 17 ± 7 was reported for transfersome lacking the vitamin and 24 ± 9 for the group treated with the commercial gel. It was concluded that this potent anti-acne effect of the adapalene transfersomal gel, together with the reduced side effects, even upon exposure to sunlight, presents a novel method of application of this treatment not only at nighttime but also during the day.

In summary, the data on transfersomal-based systems obtained from in vitro and in vivo preclinical and clinical studies have shown enhanced delivery into and through the skin of small and large molecules with various lyophilic properties. 

#### 2.4.3. Delivery Properties of Transethosomes Incorporating Various Active Molecules Investigated for Dermal and Transdermal Administration

Song described transethosomes as phospholipid vesicular systems that combine the skin delivery properties of transfersomes and ethosomes. These carriers were investigated in a number of preclinical studies (Table 4).

The first published work on transethosomes studied the in vivo skin deposition of voriconazole in mice back skin following 12 h application and in comparison with four other systems: classic liposomes, transfersomes, ethosomes and a propylene glycol solution. The highest drug concentrations were measured in the SC for ethosomes and in the epidermis and dermis for transethosomes [55].

Asenapine maleate is an anti-psychotic drug with an extensive metabolism following oral administration. Shreya et al. [144] aimed to improve the drug bioavailability in rats using a topically applied transfersomal carrier modified with 20% *v/v* ethanol. Findings of the in vitro skin permeation study demonstrated a 309.3 μg cumulative drug amount in the receiver after 24 h by means of the modified transfersomes, compared to only 160.0 and 132.9 μg for individual ethanol and transfersome, respectively. An in vivo pharmacokinetic study indicated a significant (*p* < 0.05) increase in the bioavailability upon transdermal application of the ethanolic transfersomes compared with oral administration. 

In a recent study by Albash et al., [64] the anti-hypertensive effect of an olmesartan medoxomil transethosomal system was evaluated in rats with elevated blood pressure. The effect of the transethosomes applied onto the skin was compared to that of oral administration of a commercial tablet. Elevated blood pressure to above 150 mmHg was obtained by subcutaneous injection of methyl prednisolone acetate. Results showed that the use of the drug incorporated in the transdermal vesicular system led to controlled blood pressure values for 24 h, with the highest percentage reduction at four hours (35 ± 12%). On the other hand, the oral drug administration led to controlled values for six hours only. 

#### 2.4.4. Delivery Properties of Glycerosomes Incorporating Various Active Molecules Investigated for Dermal and Transdermal Administration

The effect of glycerosomes on drug delivery through the skin was mainly studied on anti-inflammatory molecules such as diclofenac sodium and celecoxib (Table 5).

In their publications on glycerosomes, the authors present the in vitro permeation of diclofenac sodium through new-born pig skin following administration of three glycerosome systems containing three different phospholipids; DPPC [39], PL90H [40] and DMPC [41] in comparison with conventional liposomes containing the same phospholipids at similar concentrations. The results showed an improved in vitro skin deposition, with intense accumulation in the SC. 

An additional study described glycerosomal gel development for topical application of celecoxib and cupferron by the Box–Behnken statistical design. The in vitro application to rat hairless skin for eight hours of optimized systems containing 5 mg drug resulted in permeation values of 900.18 ± 50.24, 527.99 ± 34.90 mg.cm^2^ for celecoxib and cupferron, respectively. Furthermore, the in vivo anti-inflammatory effect of the two systems versus indomethacin 1% gel was evaluated in rats with carrageenan-induced acute inflammation. The obtained data showed a high percent inhibition of rat paw volume following the application of glycerosomal gel formulations, reaching 100% after 60 and 120 min compared to only 20% in the animals treated with the indomethacin product [147].

#### 2.4.5. Comparative Skin Penetration Studies on Drugs Incorporated in Ethosomes and in Transfersomes

Work was carried out to compare the delivery enhancement behavior between these two types of vesicular carriers [76,77,83,88,94,101,142]. Celia et al. prepared ethosomes and transfersomes containing linoleic acid as an active ingredient, using Phospholipon 100 G and ethanol (30–45%), or sodium cholate, respectively. The permeated amounts of the molecule from ethosomes, transfersomes and a hydroethanolic solution, through samples of human stratum corneum and a viable epidermis membrane obtained from abdomen skin after plastic surgery were measured over 24 h. The measured permeated amounts were 237.75, 57.66, 195.15 and 39.61 μg/cm^2^ for the three systems, respectively. The results of this study showed that the ethosomal carrier enabled a much better skin penetration than the transfersome. The authors suggested that this low permeation profile for transfersome could be due to the interaction between linoleic acid, phospholipid and sodium cholate [83].

In another study, soybean phosphatidylcholine-based transfersomes and ethosomes containing Tween 80 and ethanol, respectively, were comparatively investigated in vitro and in vivo. The in vitro evaluation of lycopene skin retention in fresh pig ear skin was shown to be significantly lower (*p* = 0.024) for transfersomes than for ethosomes after a 24 h application under non-occluded conditions. The anti-inflammatory effect of lycopene loaded in transfersomes and ethosomes was investigated in vivo on mice with anthralin-induced ear swelling. The results show that lycopene applied from ethosomes and transfersomes inhibited the ear swelling with no significant difference between the systems [88]. The same authors published another comparative study evaluating the effect of ethosomes, transfersomes and transethosomes on the penetration behavior of the lipophilic and hydrophilic molecules of vitamin E and caffeine, respectively. Twenty-four hours in vitro application to fresh pig ear skin of vitamin E transfersomes containing sodium cholate increased the molecule accumulation in the SC two- and six-fold when compared to ethosomes and transethosomes, respectively. This retention profile in SC was reversed in the epidermis and dermis layers, showing a higher, but not significant, accumulation of the active molecule from ethosomes and transethosomes. The results obtained with caffeine indicated a nonsignificant difference in the penetrated molecule amount in the SC between the three vesicular carriers. In the deeper skin layers and in the receiver fluid, a ~4-fold increase was measured for the caffeine amount applied in ethosomes when compared to transfersomes and transethosomes combining ethanol and sodium cholate [142]. From this investigation, it appears that ethosomes may better deliver this active molecule to the deeper layers of the skin when compared to transfersomes.

Transfersomes and ethosomes loaded with diclofenac sodium were studied in vitro on excised rat skin in comparison with conventional liposomes. The vesicles in the three systems are composed of 1% *w/w* soy lecithin and 0.3% *w/w* cholesterol. Span 80 (0.3% *w/w*) and ethanol (20–40% *w/w*) were used to alter the transfersomal and the ethosomal vesicles, respectively. The reported results indicated a comparable penetration-enhancing effect for transfersomes and ethosomes. The permeated amount of diclofenac was 2–3 times higher in the two systems when compared with liposomes [101]. These results may suggest that the presence of cholesterol in the vesicle structure alters the softness properties of ethosome. 

Ginsenoside Rh1 is another example of a molecule incorporated in the two vesicular carriers. Lipoid S75-3, cholesterol transfersomal and ethosomal vesicles altered with 0.5–1% *w/w* Tween 80 and 20% *w/w* ethanol, respectively, were evaluated in an in vitro experiment on human cadaver skin. Permeation results indicated that transfersomes led to a three-fold higher accumulation in the receiver compartment of the diffusion cell than ethosomes [94]. Again, the relatively reduced delivery behavior across the skin of ethosomes loaded with ginsenoside Rh1 can be related to the presence of cholesterol in the vesicles, affecting the fluidity of the phospholipid vesicles. 

### 2.5. Safety Assessement for Systems Containing Altered Phospholipid Vesicles for Dermal and Transdermal Delivery 

**Figure 6:**
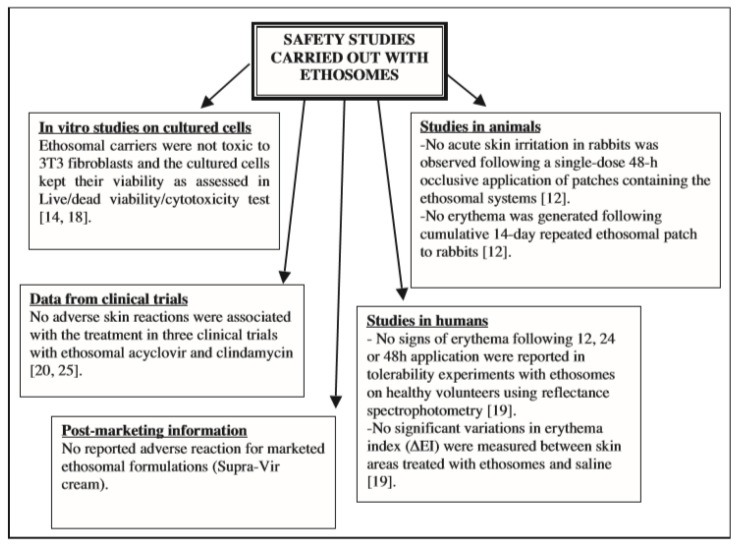
Safety studies carried out with ethosomes. Reproduced from Reference [24] with permission.

The safety of ethosomal systems has been tested in numerous in vitro and in vivo studies on cultured skin cells, in animals and human clinical studies, and on marketed products [4,34,46,70,78,79,106]. Most of these studies are schematically illustrated in Figure 6 [24]. 

In the clinical study carried out on patients with acne vulgaris, the combination of salicylic acid and clindamycin in ethosomal gel was well tolerated. No severe or moderate skin adverse effects were related to the ethosomal treatment. In addition, 82% of the participants reported that the ethosomal gel caused less erythema, burning, pruritus and photosensitivity reactions compared to commercial topical medications [79]. Paolino et al. [70] examined skin tolerability of ammonium glycyrrhizinate ethosomes vs. saline and a water–ethanol (55:45) solution as control systems in a study on healthy volunteers. The results indicated an absence of erythema signs following 12, 24 or 48 h of application of the ethosomal carrier. Recently, Ma et al. [106] investigated in vivo the potential irritant effect of paeonol incorporated in ethosomes. Histopathologic examination of skin cross-sections excised from rats exposed occulsively to the ethosomal system for 24 h showed no signs of inflammation or irritation. The tissue was normal and similar to that excised from untreated animals. 

The results of these studies indicated the safety and non-irritant effects of ethosomal carriers to skin cells and structures. 

Transfersomes were evaluated for their tolerability and skin irritation effects. Gupta et al. [121] carried out a skin irritation test on guinea pigs following 7 days of application of sertraline transfersome composed of soy lecithin and span 80. No erythema or edema, as scored visually, were found on the guinea pigs’ bare skin after applying the drug transfersomal formulation. In another in vivo investigation on rats, topical application for 24 h of eprosartan mesylate transfersomes, based on PL90G and Tween 80, was found to be safe and non-irritant [133]. Raj et al. [129] conducted visual and histopathological skin irritation tests upon the application of cytarabine nanodeformable liposomes containing SPC and sodium deoxycholate to rats for 10 days. Notably, topical application of cytarabine solution caused strong erythema. As reported by the authors, this side effect of the drug was much weaker when administrated in the transfersomal system. Such an effect could be due to higher penetrability of nanodeformable liposomes, which probably minimized the drug contact with the skin surface.

The safety of ketoprofen transfersomal gel was assessed as part of a 12 week randomized study of topical therapy application of the system to patients with knee osteoarthritis. Erythema, the most frequent dermal adverse event, was reported to affect 8.6, 8.1 and 3.6% of the participants who received ketoprofen at the doses 100, 50 and 25 mg from transfersomal system, respectively. An empty carrier caused erythema in 4.5% of the patients. Other adverse effects including skin irritation, dry skin, eczema, contact dermatitis, pruritus, and rash, were reported by 1−5% of the patients in all the treatment groups. Most of the cases were mild or moderate and were resolved without action. Those adverse reactions requiring intervention were resolved by applying ointment, cool packs, or cream [122].

## 3. Phospholipid-Altered Nanovesicles for Nasal Drug Administration

To achieve efficient nasal drug delivery, some strategies may be applied to overcome the permeability barriers in the nasal mucosa. Mainly, three aspects can be considered in the design of nasal delivery systems: the drug, the delivery carrier, and the administration device. These factors were schematically presented as a three-lobe fleur-de-lys by Touitou and Illum [12].

Nanoparticles, colloidal systems and surfactants have been used to improve the brain absorption of active compounds such as tacrine, insulin, and human growth hormones [148,149]. Vesicular carriers such as liposomes and niosomes based on amphiphilic compounds have also been investigated for nasal delivery to the brain [150]. Recently, the approach of using modified phospholipid vesicles has received significant attention [8,11].

### 3.1. Types of Phospholipid Vesicular Carriers for Nasal Administration: Properties and Mechanism of Action

#### 3.1.1. Phospholipid Nanovesicles Containing Ethanol and Propylene Glycol for Nasal Drug Delivery

Touitou et al. [8] designed and investigated the first carrier containing phospholipid vesicles altered with ethanol and propylene glycol for nasal drug delivery to the brain and systemic circulation. 

**Figure 7:**
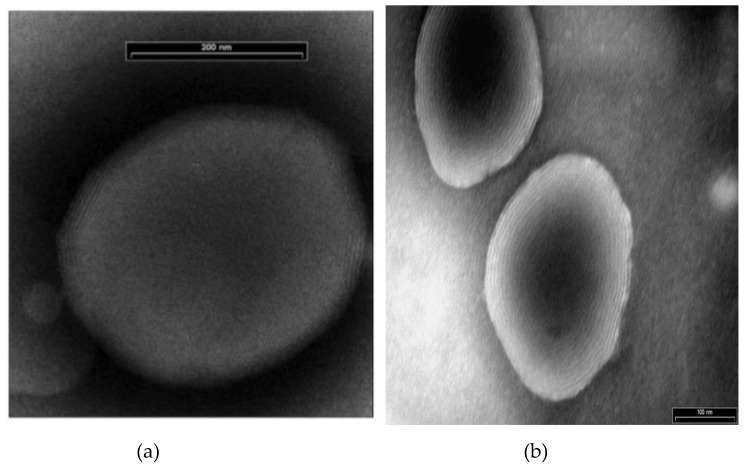
TE micrographs showing the multilamellar structure of: (**a**) Soft vesicular nasal nanocarrier, ×110k, scale bar 200 nm. Reproduced from Reference [10] with permission. (**b**) Phospholipid magnesome ×135k, scale bar 100 nm. Reproduced from Reference [152] with permission.

The carrier is prepared at room temperature using a mixing method, dissolving phospholipid in ethanol, to which propylene glycol is added. An aqueous solution of the drug is then added to the solvent solution through mixing with an overhead stirrer. Electronic microscopy of the carrier indicated the presence of multilamellar spherical nanosized vesicles (Figure 7a) with a mean size distribution of 209.7 ± 27.5 nm as measured by dynamic light scattering (DLS). Results of DSC measurements have indicated a difference of 16.51 °C in the Tm of the phospholipid vesicles of this nasal nanocarrier when compared to classic liposomes. These data suggest the presence of vesicles with fluid phospholipid bilayers in the nanocarrier [10]. 

The drug delivery-enhancing properties of this carrier were tested in several studies. Touitou et al. [10] measured the ability of soft phospholipid vesicles containing 15% *w/w* ethanol and 20% *w/w* propylene glycol to increase the in vivo penetration into rats’ nasal mucosa of fluorescent probes. CLSM data showed enhanced penetration of three probes: FITC, FITC–bacitracin and rhodamine B into the nasal mucosa only from the nanovesicular carrier, and not from a simple solution. It was hypothesized that the fluidity of the vesicles contributed to this enhanced penetration behavior.

Drugs loaded in this carrier were efficient for the treatment of various diseases in animal models, including multiple sclerosis (MS), hot flushes, pain, inflammation, migraine and insomnia [9,10,31,151].

Recently, another nasal carrier containing soft phospholipid vesicles, phospholipid magnesome, was presented by Touitou and Natsheh [152]. The carrier is composed of phospholipid, propylene glycol, magnesium ion, water and optionally a mucoadhesive polymer such as alginate salt. It is prepared by a mixing method, where the phospholipid is dissolved in propylene glycol, which is then followed by the addition of the other components [11]. The carrier examination by TEM, SEM and cryoSE microscopy indicated the presence of multilamellar vesicles (Figure 7b). DLS data showed vesicles with a mean size distribution of 739.8 ± 89.6 nm. The entrapment capacity of tramadol in the vesicles was found to be 71.2 ± 3.5% as calculated from the ultracentrifugation and HPLC results. DSC data revealed a Tm value of −7 °C for the phospholipid in the magnesome vesicles vs. +7 °C for liposomes. Compared with classic liposome, this Tm value, lower by 14 °C, points towards fluidization of the phospholipid lamellae in the magnesome vesicle.

**Figure 8:**
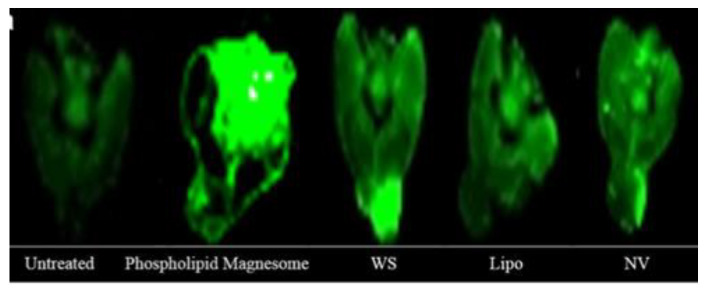
Delivery of epidermal growth factor (EGF) to the brain; near infrared imaging (NIR) image of brain of mice treated with 1 mg/kg EGF IRDye^®^ 800CW incorporated in phospholipid magnesome and in three control systems: water solution (WS), liposome (Lipo) and nonvesicular carrier (NV). Reproduced from Reference [152] with permission.

Phospholipid magnesome was designed to enhance the nasal delivery of peptides, proteins, and small molecules to the brain. The authors reported that the system containing 3% phospholipid, 15% propylene glycol, 0.1% magnesium sulfate and 0.6% sodium alginate per weight enabled enhanced nasal delivery of rhodamine 6 G (R6G), insulin, and epidermal growth factor (EGF) to various brain regions. An improved brain delivery of up to eight-fold was achieved for these molecules following their administration in the new carrier when compared to three other nasal carriers: a water solution (WS), liposome (Lipo) and a nonvesicular carrier (NV). The results were obtained from examination by multiphoton microscopy of the brain of mice treated with R6G or insulin–FITC, and by near infrared imaging (NIR) for animals that received EGF-IRDye^®^ 800CW. NIR brain imaging showed a high accumulation of the molecule in the cerebrum and in the olfactory bulb, 10 min after nasal administration in the new carrier (Figure 8). The calculated fluorescent signals were 17.6 ± 2.5 A.U. for the mice group treated with phospholipid magnesome system, in comparison with 10.0 ± 1.37, 6.7 ± 3.4, and 4.7 ± 1.0 A.U. for WS, Lipo, and NV, respectively. The obtained data suggest a possible direct nose to brain pathway and indicate rapid and enhanced delivery to the brain of the tested molecules when administrated from this vesicular carrier [152]. 

#### 3.1.2. Phospholipid Nanovesicles Containing Surfactants for Nasal Drug Delivery

Transfersomal systems containing phospholipid nanovesicles modified with edge activators used for nasal drug administration are similar to those designed for transdermal delivery in terms of components, preparation method and vesicles elasticity and deformability. These carriers are mainly obtained by a thin layer evaporation method. Salama et al. [153,154] designed two systems for nasal delivery of olanzapine to the brain based on phospholipid and edge activators. Transfersomes based on phosphatidylcholine as the lipid matrix and surfactants, including sodium deoxycholate, Span 60, Cremophor EL, Brij 58, and Brij 72, were formulated by the authors. In this work, the presence of a possible direct correlation between the elasticity of the vesicle and the amount of drug reaching the brain was investigated. Results of vesicle-elasticity tests indicated that phosphatidylcholine combined with sodium deoxycholate or Span 60 at a molar ratio of 10:1 formed vesicles with the highest deformability. These vesicles were spherical with a diameter of around 400 nm and a high deformability index. Results of the deformability studies indicate an increase in vesicle deformability with a decreasing phospholipid/ edge activator molar ratio from 100:1 to 10:1. The authors suggested that this might be attributed to fluidization of the lipid bilayer. This was further confirmed by the DSC thermogram of the unloaded transfersomes which revealed a shifting of the sodium deoxycholate endothermic peak from 336.0 to 299.20 °C and disappearance of the phospholipid peak. In transfersomes with Span 60, the peaks of both phospholipid and the surfactant disappeared [153]. 

In their second publication, phosphatidylcholine colloidal vesicles with a predominant cubic shape were obtained by incorporating the non-ionic copolymer, poloxamer 188, at a molar ratio of 10:1. The mean diameter of the vesicles was 363–645 nm. The structures in the optimized nasal system were shown to have a higher elasticity, reaching almost double the value of that of liposomes. DSC thermogram for the unloaded cubic nanovesicles revealed a shifting of 4.65 °C of the phospholipid peak from 14.88 °C in conventional liposomes to 10.24 °C, indicating the presence of the phospholipid in a liquid crystalline state [154]. It is important to mention that these differences of less than 5 °C in Tm values are much lower than those reported to the fluidizing agents: ethanol and propylene glycol, where the difference was three-fold higher. These results sustain the difference between a “soft” and an “elastic” vesicle. 

Aboud et al. [155] prepared carvedilol transfersomes by incorporating different edge activators, including Span 20, Span 60, Tween 20, Tween 80, and sodium deoxycholate, in phosphatidylcholine at different ratios. Transfersomes prepared with 95:5% (*w/w*) (phosphatidylcholine: Span 60) showed the highest entrapment efficiency of 88.72% and a mean vesicles diameter of 401 ± 5.7 nm. 

**Figure 9:**
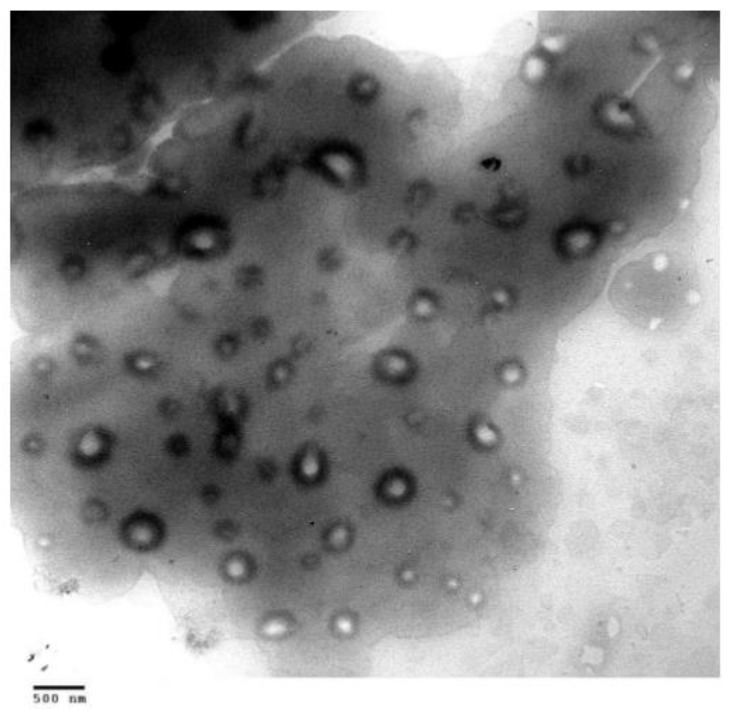
Transmission electron micrograph of chitosan composite transfersomes showing the outline and the core of spherical vesicles, ×20,000, scale bar 500 nm. Reproduced from Reference [156] with permission.

Mouez et al. [156] proposed transfersomal vesicles tailored in a mucoadhesive system for enhanced nasal drug delivery. Chitosan composite-transfersomal vesicles containing verapamil were prepared. Span80, Tween 80, sodium deoxycholate, oleic acid, Labrasol and Transcutol were used as edge activators in these systems. TE microscopic examination of the composite composition containing sodium deoxycholate revealed the presence of spherical vesicles with a thick outline and a hollow core (Figure 9). Chitosan composite vesicles displayed a larger particle size than regular vesicles lacking the polymer. For example, microparticulate systems prepared with Span 80 were found to have a mean particle size of 2004 ± 217nm compared to 1120 ± 53 nm for the corresponding vesicles lacking chitosan. This could be due to the coating potential of chitosan on the vesicular bilayer as displayed by TEM.

#### 3.1.3. Phospholipid Nanovesicles Containing Glycerol for Nasal Drug Delivery

Recently, the use of glycerosomes for nasal drug administration has been evaluated. Lacidipine glycerosomes were prepared using a thin layer evaporation technique and optimized using the Box–Behnken design method by Naguib et al. [157]. The formulation containing PL90, cholesterol and glycerol (4.58, 0.1 and 40% *w/v*, respectively), contained spherical nanovesicles with a mean size distribution of 220 nm and an entrapment efficiency of 61.97%. Elasticity examination of the vesicles by the extrusion method indicated a high deformability index of 93.65 ± 6.72.

### 3.2. Mechanism of Action of Altered Phospholipid Nanovesicles for Nasal Delivery of Active Molecules

The enhanced delivery of drugs from the nasal cavity by phospholipid nanovesicles altered with alcohols may be governed by a mechanism similar to that of ethosomes. Ethanol and propylene glycol seem to play a double fluidizing role on the vesicles’ bilayers and on the lipid bilayers in the nasal mucosa. Thus, the disrupted membrane allows for the penetration of the soft vesicles to diffuse and release their content in the deeper mucosal layers. 

The exact mechanism of action and the interactions between phospholipid magnesome and the nasal mucosa are not yet elucidated [152]. Natsheh and Touitou suggest that several concomitant processes might take place following the nasal application of the phospholipid magnesome-containing drug. On one side, the soft nanovesicles enhance drug permeation through the nasal mucosa by fluidizing the membrane lipids. In addition, the alginate matrix may interact with the mucus layer covering the nasal mucosa, slowing down the mucocilliary clearance. This process may allow a longer residence and penetration time for the drug incorporated in the vesicles. The active molecules are delivered from the nasal cavity to different areas in the brain through the olfactory region and trigeminal nerve pathways.

The proposed mechanism of action of transfersomal vesicles for nasal drug administration is based on the vesicle elasticity and deformability that enable them to squeeze through the nasal mucosa. It is also suggested that these vesicles increase drug permeation by disrupting the mucosal membrane and thus opening “new pores” in the paracellular tight junctions. In addition, nonionic surfactants such as Span 60 and poloxamer 188 may overcome the mucus barrier in the nasal mucosa by reducing its viscosity [153,154]. The combination of these nanovesicles with other penetration enhancers, such as chitosan, was reported to lead to a better penetration through the nasal mucosa. CLSM data obtained by Mouez et al. [156] suggest that the addition of chitosan to verapamil transfersomes slowed the drug in vitro release but managed to achieve high and deep penetrability across sheep nasal mucosa. These findings suggest a synergistic effect of the mucoadhesive penetration enhancer, chitosan, and the flexible transfersome vesicle. 

### 3.3. Active Molecules Incorporated in Phospholipid-Altered Vesicles Investigated for Nasal Administration

Phospholipid nanovesicles modified by alcohols for nasal administration were evaluated in several pharmacodynamic and pharmacokinetic preclinical studies. Drugs loaded in these carriers were investigated for the treatment of various diseases in animal models, including multiple sclerosis (MS), hot flushes, Parkinson’s disease, pain, inflammation, migraine and insomnia. Drug-loaded transfersomal nasal carriers were tested in a number of pharmacokinetic studies in animals. Table 6 summarizes the active molecules incorporated in these carriers and their applications.

The first published work on a nasal vesicular nanocarrier investigated its effect on tramadol delivery and analgesic activity in mice. This delivery system was shown to be effective for analgesia with a quick onset of action when compared to a nasal aqueous solution. High plasma concentrations were achieved 10 min after treatment confirming a rapid systemic absorption of the drug [31].

**Figure 10:**
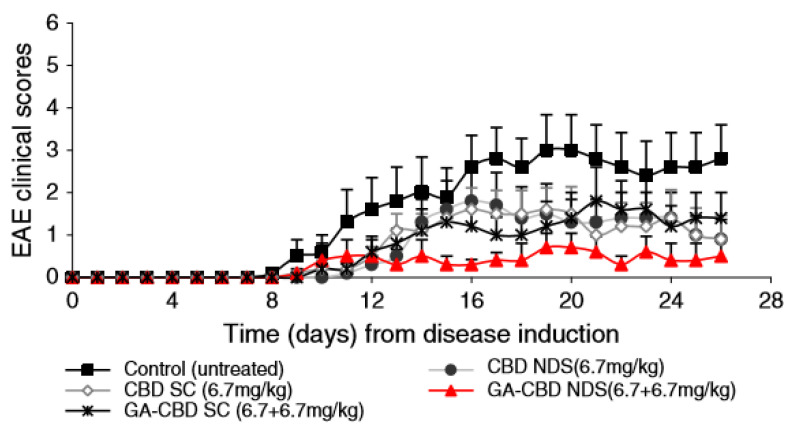
Mean clinical scores (mean ± SE) in EAE mice that received 6.7 mg/kg/days CBD alone or in combination with 6.7 mg/kg/day GA nasally from a nasal nanovesicular carrier (NDS) or subcutaneously, starting from the first day of the clinical manifestation of the disease. Reproduced from Reference [151] with permission.

Important results were obtained with this carrier in the delivery of drugs for noninvasive treatment of MS in an animal model. In this work a new drug combination was proposed by the authors, glatiramer acetate (GA), a peptide molecule, and cannabidiol (CBD). They were administrated in the nanovesicular carrier and the treatment effect was evaluated in the experimental autoimmune encephalomyelitis (EAE) mice. As reported by the authors, this treatment resulted in a statistically significant reduction in clinical scores and the inflammatory cytokine expression (Figure 10). Furthermore, a neuronal regeneration in the hippocampus of EAE mice was observed [151].

In a recently published work on the nasal nanovesicular carrier, tramadol-containing systems were tested for their analgesic effect in animal pain models. Efficient analgesia expressed by maximum possible effect (MPE%) values of more than 60% were obtained in mice acetic acid pain models, while, in comparison, the value MPE did not exceed 35% in other nasal delivery carriers and oral treatments. This analgesic effect started 10 min after treatment and lasted for the three-hour experiment period. These promising data were further confirmed by the results of a pharmacodynamic evaluation in rat pain model and sustained by tramadol-measured plasma and brain levels. Furthermore, the behavior of tramadol nasal nanosystem was measured in larger animals, sheep, in a pharmacokinetic crossover experiment that compared it with i.v. bolus administration. In this experiment, rapid and efficient delivery of tramadol was measured in plasma and cerebrospinal fluid (CSF) with calculated absolute bioavailability values of 1.09 and 0.87, respectively. Levels of *O*-desmethyl tramadol (M1), the main active metabolite of tramadol, were also measured in sheep plasma. As detected by liquid chromatography-mass spectrometry (LC-MS/MS), M1 plasma concentration in animals treated with tramadol nasally was similar to the levels in those receiving the drug by i.v administration [10].

Work with magnesome systems using multiphoton microscopy and NIR imaging of mouse brains showed effective delivery of the tested molecules to the brain following nasal administration when compared to controls. The anti-nociceptive effect of oxytocin magnesome was measured in a pharmacodynamic study on mice acetic acid pain model, and was compared to the other three control systems. Quick onset of action was observed 5 min after oxytocin phospholipid magnesome administration with an MPE of 63.5%, a considerably higher value than the values of 35.3, 30.8 and 29.9 for the controls of a water solution (WS), liposome (Lipo) and a nonvesicular carrier (NV), respectively. During the next two hours of the experiment, the effect of phospholipid magnesome was maintained, while other carriers failed to achieve a proper analgesia exhibiting MPE values of less than 30%. The results of this study suggest that a phospholipid magnesome nasal carrier can rapidly improve the effect of centrally acting active molecules [152].

The use of phospholipid vesicular carriers incorporating surfactants has been evaluated in a number of pharmacokinetic studies. Salama et al. [153,154] published two studies on these systems for nasal delivery of the anti-psychotic drug, olanzapine, to the brain. In the first work nasal administration of olanzapine from nanocubic vesicles containing poloxamer 188, to rats, resulted in 100% brain-targeting efficiency of the drug, compared to only 80% for liposomal vesicles. The absolute bioavailability of olanzapine administrated from the nanocubic vesicles was 37.9%, compared to only 14.9% for liposome [153]. In a second study by the same author, the delivery to plasma and brain of rats was investigated following nasal treatment with olanzapine loaded in two transferosomes incorporating sodium deoxycholate and Span 60, and was compared with an i.v.-administrated drug solution. The reported absolute bioavailability values in rat plasma for the two vesicular systems were 24.75 and 51.35%, respectively. The AUC_0–360_ values in rat brain were 22,334.6 ng/mL.min for sodium deoxycholate transfersomes and 36,486.3 ng/mL.min for Span 60-containing vesicles [154].

The zolmitriptan transfersomal system, prepared with soy lecithin and Tween 80, was tested in in an in vivo pharmacokinetic study in rats, and was compared to the marketed nasal spray (Zolmist^®^). The measured plasma drug levels in the animal group treated with the vesicular system showed Cmax, AUC_0–10_, MRT and t_1/2_ values of 3.60 ± 0.26 mg/mL, 11.49 ± 0.82 mg/mL.h, 4.77 ± 0.17 h, 3.45 ± 0.18 h, respectively. The marketed nasal spray (Zolmist^®^) yielded lower values of all four pharmacokinetic parameters (2.20 ± 0.16 mg/mL, 6.68 ± 0.35 mg/mL.h, 4.40 ± 0.14 h, 2.91 ± 0.11 h, respectively). The bioavailability of the transfersome formulation was found to be 1.72 times higher than that of the marketed product, suggesting efficient brain targeting by the transfersomal carrier [158]. 

The anti-hypertensive drug, carvedilol, was loaded in an in-situ gelling transfersomal formulation and its ability to enhance the drug systemic bioavailability via the nasal route was tested in rabbits. The results indicated that transfersomes enhanced carvedilol permeation through the nasal mucosa, achieving a mean Cmax of 157.47 ± 3.45 ng/mL, while the drug oral suspension achieved a mean of only 57.02 ± 4.38 ng/mL. The reported absolute drug bioavailability for the drug loaded in the nanovesicles was higher than that for the oral suspension (63.4% versus 24.1%), indicating efficient nasal delivery to the systemic circulation via transfersomes. The authors attributed this improved bioavailability to the avoidance of first pass effect and the increased permeability due to the nanotransfersomal carrier, which acted as a penetration enhancer [155].

In another work, Muoez et al. [156] showed a superior effect of chitosan-transfersomal vesicles loaded with verapamil, with an absolute plasma bioavailability of 81.83% in rabbits. Regular transfersomes prepared with soybean phosphatidylcholine and sodium deoxycholate showed a significant, but less potently enhanced nasal delivery of verapamil. The absolute bioavailability of the drug administrated to rabbits from transfersomes was found to be 65.74%, compared to 47.84% for the nasal aqueous solution and 13.04% for oral verapamil administration. This high efficiency of chitosan-transfersomal vesicles show the effect of combining penetration enhancers.

Glycerosomes were first investigated for nasal administration of the anti-hypertensive drug lacidipine. This system was evaluated in methylprednisolone acetate-induced hypertensive rats in comparison with an oral suspension of the drug. The results indicated a marked gradual reduction in blood pressure values towards the normal range, with a maximum effect at four hours continuing up to 24 h (*p* < 0.05 compared to untreated hypertensive group and *p* > 0.05 compared with normotensive rats). The administration of lacidipine oral suspension led to a slight and nonsignificant reduction (*p* > 0.05) in the blood pressure values with a maximum effect after two hours, followed by a gradual rise in blood pressure values [157].

In summary, nasal carriers based on modified phospholipid nanovesicles were shown to be much more efficient in delivering drugs when compared to conventional liposomes. The nasal administration of active molecules loaded in phospholipid nanovesicles modified by ethanol and propylene glycerol led to enhanced delivery to the brain and systemic circulation. In addition, these treatments were shown to be efficient for the management of various diseases in animal models. Transfersomal nasal systems led to enhanced systemic and brain absorption, as shown in number of pharmacokinetic studies in animals. Transfersomes nasal system were not tested for disease treatment in animal models. The rapid and efficient nasal delivery of drugs to the brain and to systemic circulation was achieved by means of these novel carriers.

### 3.4. Safety of Phospholipid Vesicular Carriers for Nasal Drug Administration

The local safety reports of sub-chronic administration of the nasal nanovesicular carrier containing ethanol and propylene glycol indicated its tolerability and nonirritant effect during the tested periods. Systems incorporating glatiramer acetate, buspirone and tramadol were administrated to rats for 14 consecutive days. The histopathological examination of treated nasal cavity sections indicated the absence of pathological changes and inflammation signs. The tissues appeared normal with no difference from those excised from healthy untreated animals. An intact and ciliated mucosal epithelium, an empty lumen and absence of infiltration or inflammation, integrity of the turbinate bone and of cartilage were observed in tissues excised from animals treated with various nasal nanovesicular systems [9,10,151].

The local safety of phospholipid magnesome was assessed upon twice-a-day administration for one week. The micrographs of the nasal cavity and nasal mucosa for phospholipid magnesome were identical to those of the untreated control group. The mucosal epithelium was found to be intact, ciliated and normal with an empty lumen. In addition, no inflammatory cells infiltration was observed, suggesting the tolerability of the carrier for the tested period. A positive control group treated with sodium lauryl sulfate exhibited minimal proteinaceous material in the lumen and focal aggregations of neutrophils [152].

Since transfersomes contain surfactants, it is necessary to consider their potential nasal mucosal irritation. As published by Salama et al. [153], light photomicrographs taken from anterior cross-sections of the nasal cavities of rats after 14 days of exposure to olanzapine transfersomal formulations (containing sodium deoxycholate and Span 60 as edge activators) showed mild-to-moderate reversible inflammation of the nasal epithelium associated with a number of inflammatory cellular infiltration, edema, and congested blood vessels. Severe inflammation signs, such as appearance of necrosis, sloughing of epithelial cells, or hemorrhage, were not detected in the tested tissue. Similar observations were reported by other studies on transfersomes. Mild congestion of blood vessels and the presence of few infiltrations of inflammatory cells were reported when rats were treated with olanzapine nanocubic vesicles containing poloxamer 188 [154]. Light photomicrographs of anterior cross-sections of rats’ nasal cavities treated for 14 days with in situ gelling transfersomal formula containing carvedilol, showed moderate degenerative changes in the olfactory epithelium and moderate mononuclear cell infiltrations in the lamina propria [155]. 

Mouez et al. reported different data on the safety of transfersomes on the nasal mucosa. A histological examination of rabbit nasal mucosa following 21 days administration of verapamil-loaded chitosan composite transfersomes comprising sodium deoxycholate showed a normal ciliated respiratory epithelium and normal goblet cells with no signs of histological alternations [156].

These controversial data regarding the toxicity and tolerability of transfersomes for nasal administration urge the need for further evaluation of their safety on the nasal mucosa. 

The toxicity of optimized lacidipine glycerosomes was assessed in vitro compared to in PBS two hours after application on isolated sheep nasal mucosa. Photomicrographs of the treated tissue showed no pathological alterations, with intact lining epithelia of both anterior and posterior segments. Normal hair follicles, sebaceous glands, and blood vessels were present in the underlying submucosa. The authors showed that nasal treatment with isopropyl alcohol for the same period caused remarkable histological alterations, where vascular proliferations with perivascular inflammation were observed in the anterior mucosal segment, and metaplasia of the mucosal lining of the epithelium with submucosal inflammation in the posterior part [157]. 

## 4. Conclusions

We covered here a large number of publications on different phospholipid vesicular carriers for enhanced delivery of active molecules for dermal/transdermal and nasal administration. Our aim was to focus on the difference between the main characteristics, preparation methods and administration modes of the altered phospholipid vesicles. 

Transferosomes, phospholipid elastic and deformable nanovesicles, modified by surfactant edge activators which enable the vesicles to penetrate through skin pores much smaller than their own size. In contrast, ethosomes, vesicles altered by the presence of high concentrations of ethanol, are soft phospholipid nanovesicles. In this carrier, the alcohol plays two major roles, fluidization of phospholipid vesicle bilayers and of the lipids in the SC, allowing the vesicles to penetrate deeper into the skin. Ethosomes can enhance skin permeation following either occlusive or non-occlusive application on the skin. A non-occlusive application of transfersomes is mandatory to achieve sufficient drug penetration into the SC. Transethosomes and glycerosomes were also studied for enhanced delivery of molecules into and across the skin. 

Active ingredients for dermal/transdermal treatment of a wide variety of skin diseases and systemic conditions incorporated in these carriers resulted in promising results in numerous in vitro, in vivo and in clinical studies. Various tests showed that these carriers are safe and well tolerated. The efficacy and tolerability of these carriers make them suitable candidates in the development of new products for dermal and transdermal administration. 

The use of soft and flexible phospholipid vesicles for nasal drug administration is now a growing topic of research and, to date, it is limited to a number of in vitro and in vivo preclinical studies. While sub-chronic nasal administration of carriers based on soft carriers was shown to be locally safe and non-irritant, the data in this regard about vesicles containing edge activators are controversial. It is important to emphasize the need to substantially investigate the safety of these nasal carriers, as the publications in this field will likely increase in number in the next few years.

This review will guide and help researchers in their choice of a suitable carrier in the design of new systems for dermal or nasal administration. 

## Figures and Tables

**Table 1:** The effect of ethanol, edge activator and glycerol on the Tm of phospholipid in various vesicular carriers for dermal/transdermal administration.

Carrier for Dermal/Transdermal Administration	Lipid	Alcohol/Edge Activator	Active Molecule	∆Tm (°C) *	Ref.
**Effect of alcohol**					
Ethosomes	PL90G	Ethanol	-	21.55	[4]
	PL90 G	Ethanol	Bacitracin	21.5	[33]
	PL90G	Ethanol	Erythromycin	21.5	[34]
	PL90G	Ethanol	Trihexyphenidyl	24.6	[35]
	PL90G	Ethanol	Ibuprofen	26.43	[36]
**Effect of edge activator**					
Liposome with edge activator	DPPC	Tween 80	-	0.84	[37]
	DPPC	Span 80	-	7.3	[37]
	DPPC	Oleic acid	-	2.1	[37]
Deformable liposomes	PL100H	KG	Methotrexate	1.1	[38]
**Effect of glycerol**					
Glycerosome	DPPC Cholesterol	Glycerol	Diclofenac sodium	1.31	[39]
	PL90H Cholesterol	Glycerol	Diclofenac sodium	0.5	[40]
	DPMC Cholesterol	Glycerol	Diclofenac sodium	0.51	[41]

* Compared to classic liposome containing the same concentration of lipids and active molecules. PL90G—phospholion90G; DPPC—dipalmitoylglycerophosphatidylcholine; PL90H—hydrogenated soy phosphatidyl choline; DMPC—1,2-dimyristoyl-sn-glycero-3phosphatidycholine; KG—potassium glycyrrhizinate.

**Table 2:** Active molecules incorporated in ethosomes investigated for dermal/transdermal administration: in vitro, in vivo preclinical and clinical studies (presented in chronological order).

Active Molecule	Molecular Weight (g/mol)	Log p	Indication	Study	Ref.
Caffeine	149.2	−0.1 ^1^	Improving microcirculation	In vitro permeation studies on mice skin	[7]
Acyclovir	225.2	−1.2 ^2^	Anti- Herpes Simplex Virus (HSV)-1	Tow-armed double-blind clinical study on subjects with recurrent herpes labialis (RHL); anti-viral activity against HSV-1 by plaque reduction assay in monolayer cultures of Vero cells	[73,74,75]
Trihexyphenidyl HCl	337.9	4.93 ^2^	Anti-Parkinson’s	Deposition and permeation study on dorsal nude mice skin	[35]
Testosterone	288.42	3.32 ^3^	Testosterone replacement therapy	In vitro permeation through rabbit pinna skin; in vivo transdermal delivery in rabbits	[4,42]
Minoxidil	209.25	1.24 ^3^	Treatment of hair loss	In vitro penetration and permeation through abdominal nude mice skin	[4]
Cannabidiol	314.46	6.33 ^2^	Anti-inflammatory	In vivo permeation and organ distribution in mice; evaluation of the reduction in paw thickness in carrageenan-induce edema	[44]
Bacitracin	1422.69	−2.9 ^2^	Anti-bacterial	Intracellular penetration and localization in fibroblasts (3T3); in vitro deposition and permeation through human cadaver skin	[33]
Azelaic acid	188.22	1.37 ^2^	Bacteriostatic, anti-keratinizing	In vitro studies of diffusion and release using synthetic membranes	[71]
Erythromycin	733.93	2.6 ^2^	Anti-bacterial	In vivo activity in mice models of deep dermal S. aureus infection	[34]
Ammonium glycyrrhizinate	840	2.84 ^2^	Anti-inflammatory	In vitro permeation through human skin; clinical study to evaluate the anti-inflammatory activity in volunteers with methyl nicotinate erythema	[70]
Ketotifenfumarate	425.5	3.2 ^2^	Anti-histaminic	In vitro permeation and deposition study on rabbit pinna skin	[76,77]
Prostaglandin E1	354.48	3.2 ^2^	Erectile dysfunction	In-office, pilot clinical study, on men with erectile dysfunction	[78]
Clindamycin and Salicylic acid	424.98 138.12	2.16 ^3^ 1.96 ^2^	Treatment of Acne vulgaris	Clinical study on the reduction in acne vulgaris and skin tolerability of the formulation	[79]
Matrine	248.36	1.6 ^1^	Anti-inflammatory	In vitro percutaneous permeation study on rat skin; in vivo anti-inflammatory activity in rats measured by reflection spectrophotometry	[80]
Triptolide	360.41	0.2 ^1^	Anti-inflammatory and immunosuppressive	In vitro permeation study on rat skin; in vivo evaluation of erythema reduction on rats.	[81]
9-[(2-hydroxyethoxy) methyl]guanine	343.29	N/A	Anti-HSV-1	Anti-viral activity against HSV-1 by plaque reduction assay in monolayer cultures of Vero cells	[74]
Buspirone HCl	385.5	1.95 ^2^	Hot flushes management	In vivo study on ovariectomized rats with hot flushes	[43]
Ibuprofen	206.29	3.97 ^3^	Anti-inflammatory	Measurement of drug plasma concentrations in rats; in vivo anti-pyretic effect in fevered rats and analgesic effect using tail flick test in mice	[36]
Ligustrazine	136.19	1.3 ^1^	Treatment of Alzheimer’s disease	In vitro skin permeation study on abdominal rat skin; effect on Scopolamine-induced amnesia in rats using Morris water maze test; measurement of the antioxidant enzymes activities and the levels of the biomarker of oxidative stress, malondialdehyde (MDA) in the brain of the amnesic animals	[82]
Linoleic acid	280.44	7.06 ^4^	Skin whitening	In vitro percutaneous permeation through human stratum corneum and viable epidermis membrane	[83]
Paclitaxel	853.9	3.0 ^3^	Treatment of squamous cell carcinoma	In vitro permeation study on human stratum corneum (SC); in vitro anti-proliferative effect in squamous carcinoma cells	[84]
Tacrolimus	804.02	3.3 ^3^	Immunosuppressant for treatment of atopic dermatitis	In vitro quantification of drug in different strata of rat skin; in vivo evaluation of allergic reactions suppression in mice with induced ear swelling	[85,86]
Vinpocetine	350.45	4.1 ^2^	Prevention and treatment of cerebrovascular diseases and ischemic stroke	In vitro permeation through abdominal rat skin	[87]
Lycopene	536.87	9.16 ^2^	Antioxidant	In vitro skin permeation and skin retention study; in vivo tests on anthralin-induced ear swelling in mice	[88]
Tetrandrine	622.7	5.55 ^2^	Anti-inflammatory, anti-rheumatic	In vitro penetration and permeation study on rat skin; in vivo efficacy on Freund’s complete adjuvant-induced arthritis model	[89]
Lidocaine base	234.34	2.84 ^2^	Local anesthetic	In vitro penetration study on rat skin; in vivo effectiveness using pinprick test on guinea pigs	[90]
Cetirizine dihydrochloride	461.8	2.98 ^2^	Anti-histaminic	In vitro permeation and deposition study on dorsal mice skin	[91]
Apigenin	270.05	3.07 ^2^	Antioxidant and anti-inflammatory	In vitro and in vivo deposition study on rat skin; evaluation of the reduction in cyclooxygenase-2 levels in mouse with skin inflammation	[92]
Curcumin	368.38	3.62 ^2^	Antioxidant and anti-inflammatory	Clinical trial evaluating skin viscoelasticity, total deformation, biological elasticity and sagginess	[93]
Ginsenoside Rh1	638.9	4.3 ^1^	anti-allergic and anti-inflammatory	In vitro permeation study on human cadaver skin	[94]
Capsaicin	305.41	3.8 ^2^	Anti-arthritic	In vitro permeation though human cadaver skin; in vivo study on rat model of carrageenan-induced inflammation, formaldehyde-induced arthritis and evaluation of suppression of arthritis-induced hyperalgesia	[95]
Artemisinin and Febrifugine	282.33 30.1.34	2.52 ^2^ 0.07 ^5^	Anti-parasitic. Eradication of *Plasmodium spp*	In vitro transdermal penetration through abdominal mice skin; in vivo evaluation of anti-malarial activity in infected mice	[96]
Griseofulvin	352.76	2.2 ^3^	Anti-fungal	In vitro permeation and deposition study on new-born pig skin	[97]
Cryptotanshinone	296.4	3.8 ^2^	Anti-bacterial, anti-inflammatory	In vitro skin permeation and skin deposition; in vivo anti-acne activity on rabbits	[98]
Crocin	976.97	−0.02 ^2^	Antioxidant	Evaluation of the anti-inflammatory activity on healthy volunteers.	[99]
Vancomycin hydrochloride	1449.3	−2.6 ^1^	Anti-bacterial	In vitro skin permeation study In vivo evaluation of anti-bacterial effect in rats with S. aureus induced-mediastinitis	[100]
Diclofenac sodium	318.1	0.7 ^4^	Anti-inflammatory	In vitro permeation study on rat skin; in vivo anti-inflammatory activity in carrageenan-induced rat paw edema model; in vitro permeation and deposition studies on rat skin	[45,101]
Methoxsalen	216.19	1.7 ^3^	Improving melanin production, against vitiligo	Ex vivo release studies and photo- toxicity after exposure to UV light	[102]
Psoralen	186.16	2.3 ^1^	Anti-bacterial, agent in photodynamic therapy	Photodynamic therapy in biofilms formed in Petri dishes	[103]
Clove oil	164.2	2.66 ^2^	Anti-fungal and anti-bacterial	Ex vivo permeation studies on rat skin; anti-fungal activity in cup plate test against Candida albicans	[104]
Voriconazole	349.31	1.0 ^3^	Anti-fungal	In vitro anti-fungal activity against *Aspergillus flavus* colonies; in vitro skin deposition and permeation through abdominal rat skin	[105]
Paeonol	166.18	1.98 ^4^	Anti-inflammatory, anti-diabetic, and pain-relieving	In vitro transdermal absorption and skin retention studies on rat skin	[106]
Methotrexate and Salicylic acid	454.44 138.12	1.85 ^3^ 1.96 ^2^	Psoriasis management	In vitro retention and permeation study on pig ear skin; in vivo anti-psoriatic activity in mice models with imiquimod-induced psoriasis	[107]
Thymosinβ-4(Tβ-4)	4982	N/A	Wound healing	In vitro drug release study on mice skin; in vivo pharmacokinetic and skin irritation studies on mice	[108]
Febuxostat	316.37	3.8 ^2^	Anti-gout	In vitro permeation and retention studies on rat skin; in vivo pharmacokinetic study on rabbits.	[109]
Coenzyme Q10	863.34	19.4 ^1^	Antioxidant	Vesicles uptake study in fibroblasts; cytotoxic and antioxidant effect in fibroblasts.	[110]
Anthralin	226.23	2.73 ^2^	Anti-psoriatic	Preparation, comparative evaluation and clinical assessment in psoriatic patients	[111]

^1^ Predicted values [112]; ^2^ predicted values [113]; ^3^ experimental values [113]; ^4^ experimental values [112]; ^5^ predicted value [114].

**Table 3:** Active molecules incorporated in transfersomes and other deformable liposomes investigated for dermal/transdermal administration: in vitro, in vivo preclinical and clinical studies (presented in chronological order).

Active Molecule	Molecular Weight (g/mol)	Log p	Indication	Study	Ref.
Human serum albumin Gap junction proteins	66430.0 N/A	N/A N/A	Immunogenic proteins	In vivo effect on inducing antigen-specific antibodies in mice	[50]
Insulin	5734	N/A	Anti-diabetic	In vivo measurement of hypoglycemic effect in mice and healthy human volunteers	[49]
Diclofenac sodium	318.1	0.7 ^1^	Anti-inflammatory	In vitro permeation and drug deposition studies on rat skin; in vivo deposition in target tissues studies on mice, rats and pigs	[101,115]
Triamcinolone acetonide	434.5	2.53 ^1^	Anti-inflammatory	In vivo localization in mice skin; in vivo bioactivity murine ear edema; double-blind, placebo-controlled clinical trial on healthy volunteers	[116,117]
Hydrocortisone	362.46	1.6 ^1^	Steroidal anti-inflammatory	In vivo evaluation of the anti-inflammatory effect in mice with arachidonic induced edema	51]
Dexamethasone	392.46	1.8 ^1^	Steroidal anti-inflammatory	In vivo evaluation of the anti-inflammatory effect in mice with arachidonic induced edema	[51]
Methotrexate	454.44	1.85 ^2^	Anti-cancer	In vitro permeation and deposition study on pig ear skin	[38]
Ketotifen fumarate	425.5	3.3 ^3^	Anti-histaminic	In vitro permeation and skin deposition study on rabbit pinna skin	[76,77]
Meloxicam	351.4	3.43 ^1^	Anti-inflammatory	In vitro permeation study on shed snake skin from the Siamese cobra	[118]
Ketoconazole	531.43	4.3 ^4^	Antifungal	In vitro drug release across cellulose membranes and permeation study on pig skin	[119]
Terbinafine	291.43	5.51 ^3^	Antifungal	In vitro measurement of anti-fungal effect against Dermatophyte Hyphae in samples from a clinical study.	[120]
Sertraline	306.23	5.06 ^3^	Anti-depressant	In vitro drug release study on cellophane membrane; in vitro drug permeation through rat skin; in vivo evaluation of the antidepressant activity using forced swim model test	[121]
Linoleic Acid	280.44	7.06 ^1^	Skin whitening	In vitro percutaneous permeation through human stratum corneum and viable epidermis membrane	[83]
Itraconazole	705.64	5.66 ^1^	Anti-fungal	In vitro penetration and permeation study on rat skin	[54]
Ketoprofen	254.3	3.1 ^1^	Treatment of osteoarthritis	Randomized clinical study conducted on knee osteoarthritis patients	[122]
Lycopene	536.87	9.6 ^3^	Anti-inflammatory	In vitro skin permeation and skin retention study; in vivo tests on anthralin-induced ear swelling in mice	[88]
Cisplatin Imiquimod	300 240.3	−2.19 ^2^ 2.7 ^2^	Treatment of cutaneous epithelial malignancy	In vivo skin localization and biodistribution in mice; in vivo evaluation of dermal, hepato- and nephro-toxicity in mice and rats	[123]
Cinnamic acid	148.16	2.1 ^1^	Antioxidant and anti-inflammatory	In vivo micro-dialysis study in rats	[124]
Felodipine	384.26	3.90 ^1^	Anti- hypertensive	In vitro deposition and permeation study on rat skin	[125]
Tetanus toxoid	6400	N/A	Tetanus vaccine	In vivo evaluation of protection against tetanus toxin challenge in mice	[62]
Raloxifene hydrochloride	510	5.45 ^3^	Treatment of invasive breast cancer	In vitro release through dialysis membrane; in vitro permeation though rat skin; in vitro permeation and deposition study on rat skin	[126,127]
Sildenafil	474.58	2.75 ^1^	Erectile dysfunction	In vitro permeation through synthetic nylon membrane	[128]
Ginsenoside Rh1	638.9	4.3 ^4^	Anti-allergic and anti-inflammatory	In vitro skin permeation study on human cadaver skin	[94]
Cytarabine	243.22	−2.8 ^2^	Treatment of leukemia	In vitro permeation and deposition study on rat skin.	[129]
Pentoxifylline	278.31	0.29 ^2^	Treatment of intermittent claudication and chronic occlusive arterial diseases	In vitro drug release using dialysis bags	[130]
Timolol maleate	316.42	1.44 ^3^	Anti-hypertensive	In vitro permeation study through synthetic cellulose nitrate membranes; in vitro permeation study using shaved rat skin; in vivo pharmacokinetic study in rats	[131,132]
Eprosartan mesylate	520.6	3.57 ^3^	Anti- hypertensive	In vitro permeation through rat skin; in vivo anti-hypertensive effect on rats with methyl prednisolone acetate-induced hypertension	[133]
Tocopherol	430.71	8.84 ^3^	Skin regeneration	In vitro studies on human keratinocytes cells and fibroblasts; cell viability and antioxidant activity	[134]
Ondansetron	329.82	2.4 ^1^	Management nausea and vomiting	In vitro permeation study on rat skin	[135]
Minoxidil and Caffeine	209.25 149.25	3.32 ^1^ −0.1 ^4^	Treatment of alopecia	In vivo effect on hair growth	[52]
Resveratrol	228.25	2.57 ^3^	Antioxidant	In vitro transdermal delivery analysis using the Strat-M^®^ Membrane (Merck, Darmstadt, Germany)	[136]
Human Growth Hormone	848.9	N/A	Hormone replacement therapy	In vitro penetration and permeation studies through rat skin; in vitro skin permeation and biological activity.	[137,138]
Lidocaine base	234.34	2.3 ^3^	Local Anesthetic	In vitro release study through dialysis cellulose membrane	[139]
Adapalene	412.52	6.92 ^3^	Treatment of acne vulgaris	In vitro penetration and permeation study using goat skin membrane; in vivo effect on mice with testosterone-induced acne	[140]
Retinyl palmitate	524.86	10.12 ^3^	Skin regeneration	In vitro skin penetration and fluorescent biodistribution assays on pig ear skin	[141]

^1^ Experimental values [112]; ^2^ experimental values [113]; ^3^ predicted values [113]; ^4^ predicted values [112].

**Table 4:** Active molecules incorporated in transethosomes investigated for dermal/transdermal administration: in vitro and in vivo in animal studies (presented in chronological order).

Active Molecule	Molecular Weight (g/mol)	Log p	Indication	Study	Ref.
Voriconazole	349.31	1.0 ^1^	Anti-fungal	In vitro skin permeation and deposition studies through mice skin; in vivo deposition study on mice	[55]
Vitamin E	430.7	8.84 ^2^	Skin regeneration	Permeation and penetration studies on pig ear skin	[142]
Caffeine	149.2	−0.1 ^3^	Improving microcirculation	Permeation and penetration studies on pig ear skin	[142]
Imiquimod	240.3	2.7 ^3^	Anti-cancer	In vitro skin permeation study on abdominal pig skin; in vivo permeation study on rats; in vivo skin histological examination in rats	[143]
Asenapine maleate	285.8	4.35 ^2^	Anti-psychotic	In vitro skin permeation study on rat skin; in vivo pharmacokinetic study in rats	[144]
Fisetin	155.15	2.03 ^1^	Anti-oxidative, anti-inflammatory, anti-viral, pro-apoptotic and neuroprotective ability	In vitro penetration dermatokinetic studies on rat skin	[63]
Econazole nitrate	444.7	N/A	Anti-fungal	Ex vivo skin permeation and retention studies followed by in vitro anti-fungal activity against C. albicans fungus	[145]
Fe-chlorophyllin	694.8	N/A	Anti-cancer	In vitro skin permeation and deposition studies through mice skin; in vivo evaluation of the anti-cancer effect in mice	[56]
Olmesartan medoxomil	558.6	4.46 ^2^	Anti-hypertensive	Ex vivo permeation through rat skin and shed snake skin; in vivo histopathological, pharmacodynamic and dermatokinetic studies on rats	[64]

^1^ Experimental [113]; ^2^ predicted values [113]; ^3^ predicted values [112].

**Table 5:** Active molecules incorporated in glycerosomes investigated for dermal/transdermal administration: in vitro and in vivo in animal studies (presented in chronological order).

Active Molecule	Molecular Weight (g/mol)	Log p	Indication	Study	Ref.
Diclofenac sodium	318.1	0.7 ^1^	Anti-inflammatory	In vitro penetration and permeation studies on new-born pig skin	[39,40,41]
Paeoniflorin	480.5	−1 ^1^	Anti-inflammatory	In vitro permeation experiments through excised rat abdominal skin; in vivo deposition in rat synovium	[146]
Celecoxib	381.37	3.5 ^1^	Anti-inflammatory	In vitro permeation study on rat hairless skin; in vivo anti-inflammatory effect in carrageenan-induced acute inflammation in rats	[147]
Cuperron	155.15	N/A	Anti-inflammatory	In vitro permeation study on rat hairless skin; in vivo anti-inflammatory effect in carrageenan-induced acute inflammation in rats	[147]
Fisetin	155.15	2.03 ^1^	Anti-oxidant, anti-inflammatory, anti-viral, neuroprotective, and pro-apoptotic ability	In vitro penetration study on rat skin	[65]

^1^ Predicted values [112].

**Table 6:** Active molecules investigated using phospholipid vesicular carriers for nasal administration (presented in chronological order).

Nasal Vesicular Carrier	Active Molecule	Molecular Weight (g/mol)	Log p	Indication	Study	Ref.
Phospholipid carriers modified by ethanol and propylene glycol						
Soft nanovesicular carrier	Tramadol HCl	263.381	2.71 ^1^	Analgesic	In vivo evaluation of analgesic effect; measurement of drug plasma levels in mice; in vivo evaluation of analgesic effect and measurement of drug plasma and brain levels in rats; in vivo pharmacokinetic study in sheep	[8,10,31]
	Prednisolone	360.44	1.66 ^1^	Anti-inflammatory	Effect on clinical scores and inflammatory cytokines in in vivo study on encephalomyelitis (EAE) mice	[8,151]
	Glatiramer acetate Cannabidiol	623.65 314.46	N/A 6.33 ^1^	Multiple sclerosis management	Effect on clinical scores and inflammatory cytokines in in vivo study in EAE mice	[8,151]
	Brotizolam	393.7	2.7 ^2^	Treatment of insomnia	Hypnotic effect on pentobarbitone-induced sleeping in mice	[8]
	Apomorphine	267.3	2.3 ^2^	Treatment of Parkinson’s disease	Anti-Parkinson’s effect in rats with 6-hydroxydopamine (6-OHDA) induced brain lesions	[8]
	Buspirone HCl	385.5	1.95 ^1^	Hot flushes management	In vivo effect on tail skin temperature in ovariectomized rats; in vivo pharmacokinetic study in rats	[8,9]
Phospholipid magnesome	Oxytocin	1,007.19	−2.6^2.^	Analgesic	In vivo evaluation of anti-nociceptive effect in mice	[11,152]
	Insulin	5734	N/A	Treatment of diabetes mellitus	In vivo measurement of the hypoglycemic effect in mice	[11,152]
	Ketoprofen	254.281	3.12 ^3^	Analgesic	In vivo pharmacokinetic study in rats	[11]
	Epidermal growth factor	6046	N/A	Investigated as a neuroprotective agent after spinal cord injury	Visualization of delivery to brains of mice	[11,152]
Phospholipid carriers containing edge activators						
Nanocubic vesicles	Olanzapine	312.44	3.61 ^1^	Anti-psychotic	In vivo pharmacokinetic study in rats	[154]
Transfersomal vesicles	Olanzapine	312.44	3.61 ^1^	Anti-psychotic	In vivo pharmacokinetic study in rats	[153]
	Carvedilol	406.5	4.2 ^2^	Anti-hypertensive	In vitro permeation study through sheep nasal mucosa; in vivo pharmacokinetic study in rabbits.	[155]
	Verapamil HCl	491.1	3.8 ^2^	Anti-hypertensive	Bioavailability study in rabbits	[156]
	Zolmitriptan	287.36	2.2 ^1^	Treatment of Migraine	In vivo pharmacokinetic study in rats	[158]
Phospholipid carriers containing glycerol						
Glycerosomes	Lacidipine	455.54	4.5 ^2^	Anti-hypertensive	In vitro penetration and permeation studies on sheep nasal mucosa; in vivo anti-hypertensive effect in rats	[157]

^1^ Predicted values [113]; ^2^ predicted values [112]; ^3^ experimental values [112].
